# MI-SOH: a multi-indicator feature dependency model for lithium-ion battery state-of-health Estimation

**DOI:** 10.1038/s41598-026-39986-3

**Published:** 2026-03-05

**Authors:** Shilong Zhuo, Fumin Zou, Lyuchao Liao, Xinjian Cai

**Affiliations:** 1 School of Electronics, Electrical and Physics, Fujian University of Technology, Fuzhou, 350118 China; 2 Fujian Provincial Key Laboratory of Automotive Electronics and Electric Drive Technology, Fuzhou, 350118 China; 3 Ningde New Energy Technology Research Institute, Fujian University of Technology, Ningde, 352101 China

**Keywords:** Lithium-ion batteries, State of health estimation, Multi-indicator feature, Tem-poral convolutional network (TCN), iTransformer, Metaheuristic optimization, Energy science and technology, Engineering, Mathematics and computing

## Abstract

Accurate state-of-health (SOH) estimation is vital for guaranteeing the safety and longevity of lithium-ion batteries. However, most existing methods employ static feature fusion strategies that fail to account for temporal evolution of indicator correlations throughout battery degradation, leading to compromised estimation accuracy under complex, non-stationary aging patterns. To address this gap, this study proposes MI-SOH, a multi-indicator SOH estimation model that dynamically adapts to evolving feature importance across battery lifecycle stages. MI-SOH primarily consists of four core components: (1) Multi-indicator Feature Weighting Block that employs dual-correlation analysis to adaptively prioritize health factors based on correlation patterns that reflect multi-stage degradation characteristics; (2) Temporal Pattern Extraction Block that processes these weighted features through dilated convolutions to capture multi-scale degradation dynamics; (3) Cross-Variable Dependency Modeling Block that utilizes inverted transformers to learn complex interdependencies among different health indicators throughout battery aging; and (4) Adaptive Hyperparameter Optimization Block that automatically configures model hyperparameters for optimal performance across diverse battery conditions. Extensive experiments on benchmark National Aeronautics and Space Administration (NASA) and Center for Advanced Life Cycle Engineering (CALCE) datasets demonstrate that MI-SOH outperforms current mainstream prediction approaches across diverse battery chemistries and lifecycles, achieving average Root Mean Squared Error (RMSE) of 0.00312 and 0.01126 respectively. This research advances intelligent battery management systems (BMS) by providing a practical SOH monitoring framework critical for electric vehicle safety and energy storage reliability.

## Introduction

 The global transition to sustainable energy systems has accelerated the adoption of electric vehicles (EVs), which play a crucial role in reducing carbon emissions and fossil fuel dependence. As the core energy storage component, lithium-ion batteries determine the performance, safety, and economic viability of EVs. The state of health serves as a critical indicator for battery management systems, directly impacting vehicle safety, operational costs, and battery lifecycle management. Accurately estimating the battery’s SOH is crucial for extending the lifespan of EVs and ensuring their safe and reliable operation. Currently, common methods for estimating SOH include experimental testing methods, model-based methods (including electrochemical and equivalent circuit models), and data-driven methods^1^.

Experimental testing methods primarily rely on measuring internal battery parameters such as capacity, internal resistance, and electrochemical impedance to assess SOH^2,3^. While these approaches offer high accuracy through direct physical measurements, it is costly, as these approaches require specialized equipment and controlled environments impractical for real-world applications.

Model-based methods estimate SOH by constructing mathematical representations of battery behavior, including electrochemical models and equivalent circuit models^4–8^. These approaches provide clear physical interpretations and theoretical foundations for understanding battery degradation mechanisms. However, these approaches suffer from high computational complexity due to numerous parameters and demonstrate limited capability to capture the full spectrum of battery dynamic characteristics under varying operating conditions, making them impractical for large-scale battery SOH analysis.

Data-driven methods have emerged as promising alternatives by learning the mapping relationships between measurable battery parameters and SOH without requiring detailed knowledge of internal battery mechanisms^9–18^. These approaches leverage advanced machine learning and deep learning techniques to extract degradation pat-terns from historical data. Nevertheless, most existing data-driven methods rely on single-dimensional indicators or manually selected multi-indicator combinations, with static feature weighting schemes that fail to adapt to time-varying correlations under complex aging conditions.

A critical insight emerges from analyzing existing data-driven approaches: traditional health indicators exhibit dynamic interdependencies that evolve throughout battery degradation processes, yet current methods treat them as independent variables with fixed importance. Our systematic analysis reveals that indicator correlations undergo diverse transformations across degradation stages—some pairs exhibit significant strengthening, others demonstrate substantial weakening (e.g., constant current duration vs. IC peak substantially decreases), while certain voltage-related pairs show polarity reversals from negative to positive correlation. This temporal variability necessitates adaptive mechanisms that dynamically adjust feature contributions based on degradation context, rather than the static feature fusion strategies employed by existing methods.

To address these fundamental limitations, this paper proposes MI-SOH (Multi-Indicator feature dependency modeling for SOH estimation), a comprehensive framework that integrates multi-scale temporal modeling with cross-variable dependency analysis to capture evolving correlations among battery health indicators. Unlike conventional approaches, MI-SOH employs adaptive multi-indicator feature weighting based on dual-correlation analysis, while simultaneously incorporating temporal pattern extraction through dilated-causal convolutions. The framework’s adaptive weighting mechanism is designed to accommodate the temporal variability of indicator correlations—where some pairs strengthen, others weaken, and certain relationships exhibit non-monotonic evolution across degradation stages (Sect. “Temporal evolution analysis of indicator correlations”). Furthermore, the framework integrates cross-variable dependency modeling via inverted transformers, complemented by global hyperparameter optimization using the Zebra Optimization Algorithm (ZOA). This integrated approach addresses the critical limitations of existing methods while providing a practical solution for real-world battery management applications.

Given the high-dimensional hyperparameter space of MI-SOH (8 parameters including TCN configurations, iTransformer architecture, and training settings as detailed in Table 4), we employ the ZOA for hyperparameter tuning. Unlike conventional metaheuristic algorithms that rely on fixed strategies—Particle Swarm Optimization (PSO)’s constant inertia weight^19^, Whale Optimization Algorithm (WOA)’s probabilistic spiral-search switching^20^, or Grey Wolf Optimizer (GWO) ‘s deterministic hierarchical structure^21^—ZOA features an adaptive dual-phase mechanism: a foraging phase for exploitation toward optimal solutions and a defense phase with stochastic escape strategies for exploration^22^. This adaptive flexibility is particularly advantageous for non-convex optimization landscapes where exhaustive grid search becomes computationally prohibitive (requiring 10^7–10^8 evaluations). Section “Hyperparameter optimization comparison” provides empirical validation through systematic comparison with PSO, WOA, and GWO, demonstrating ZOA’s superior convergence speed and solution quality.

The main contributions of this research are threefold. (1) We identify that existing SOH estimation methods overlook dynamic interdependencies among health indicators that evolve throughout battery degradation stages, requiring adaptive treatment rather than static feature fusion. (2) We develop a comprehensive MI-SOH framework integrating multi-indicator weighting, temporal convolutions, inverted transformers, and intelligent optimization to capture temporal dynamics and cross-variable dependencies. (3) We demonstrate superior and generalizable performance on NASA and CALCE datasets, achieving RMSE improvements of 9.12%−75.24% across baseline methods, with ablation studies and cross-dataset validation confirming each component’s contribution.

The remainder of this paper is organized as follows: Sect. “Related work” reviews related work in battery SOH estimation. Section “Methodology” presents the proposed MI-SOH methodology and its key components. Section “Experiment” describes the experimental dataset, data preprocessing procedures, evaluation metrics, and presents comprehensive results including comparative experiments, temporal evolution analysis of indicator correlations, ablation studies, and complexity analysis. Finally, Sect. “Conclusions” concludes the paper and discusses future research directions.

## Related work

### Experimental testing methods

Experimental testing methods for SOH estimation include capacity, internal resistance, and electrochemical impedance spectroscopy methods. Llerandi et al.^2^proposed a new battery health assessment method that measures the internal resistance of batteries to enable continuous online monitoring in uninterruptible power supplies (UPS). Compared to traditional methods, this system does not require dis-connecting the battery, reducing maintenance costs. Zhang et al.^3^proposed a model for estimating the health state of lithium-ion batteries, utilizing electrochemical impedance spectroscopy to analyze the lithium-titanate (LTO) anode, and selected key feature parameters such as charge transfer resistance and Ohmic resistance. The model was trained and validated using a backpropagation neural network (BPNN), showing relatively accurate SOH estimation in real driving conditions.

Although these methods offer high accuracy, they are difficult to implement. Gathering the internal parameters of the battery requires specialized and costly experimental equipment, and the parameter analysis process is complex, making them unsuitable for online estimation.

### Model-based methods

Model-based methods estimate SOH by constructing an equivalent model based on the working principles and reaction mechanisms of lithium-ion batteries. These methods include electrochemical models and equivalent circuit models^4^. Electro-chemical models provide detailed descriptions of the internal characteristics of lithium batteries, with clear physical meanings and high accuracy, making them suitable for theoretical analysis. However, these models are often overly complex, with numerous parameters and a substantial computational burden^5^. Equivalent circuit models con-sist of resistors, capacitors, voltage sources, and other circuit elements, and use various combinations to simulate the internal dynamic reactions of lithium-ion batteries^6^. These models typically involve parameter identification and state space equations to predict and estimate the battery’s states. Such methods require experimental data col-lection, such as voltage, circuit, and temperature information, to establish appropriate equivalent models and state space equations, followed by parameter identification and the use of filtering algorithms for SOH prediction. Xu et al.^7^proposed an improved particle filter (PF) algorithm for SOH prediction of lithium-ion batteries, which exhibited good adaptability and high precision in predicting the aging process of lithium batteries. Chen et al.^8^employed the Square Root Unscented Kalman Filter (SR-UKF) algorithm for SOH estimation, and experiments demonstrated that the SR-UKF algorithm outperformed traditional Kalman Filter methods.

While model-based SOH estimation methods are widely used, they require an understanding of the internal mechanisms of the battery, considering both material properties and the physical-chemical reactions, resulting in complex models with numerous parameters that have limited ability to describe battery dynamic characteristics.

### Data-driven methods

Data-driven methods estimate SOH by learning the mapping relationship between battery measurement parameters (voltage, current, temperature, internal resistance) and SOH from historical data, without requiring detailed knowledge of battery internal mechanisms^9,10^. However, their performance depends critically on the quality and comprehensiveness of measurement data^11^.

Early research primarily focused on extracting individual or limited health indicators to characterize battery degradation. Lin et al.^12^used constant current charging time (CCCT) in fixed voltage ranges as the sole feature with random forest regression, achieving average RMSE of 0.52% across 8 cells. However, the method relies on a single indicator without multi-source feature fusion. Fan et al.^13^employed 10-second relaxation voltage as input with Convolutional Neural Network (CNN), achieving average APE of 1.8% across 28 commercial batteries, but requires specialized 2-hour relaxation periods after full charge and lacks multi-indicator integration.

Recognizing that lithium-ion battery degradation exhibits diverse characteristics and temporal dependencies that single indicators cannot fully capture, researchers have progressively shifted toward multi-indicator fusion strategies. Lin et al.^14^extracted seven features from voltage, temperature, and IC curves, using Random Forest Regression (RFR) to fuse predictions from Multiple Linear Regression (MLR), Support Vector Regression (SVR), and Gaussian Process Regression (GPR). However, the method treats features with equal importance and employs static RFR weights after training, lacking dynamic adjustment across battery lifecycle stages. This limitation becomes particularly significant when indicator interdependencies evolve throughout degradation processes, as some correlations strengthen while others weaken with aging.

To better leverage complementary strengths of different deep learning architectures, recent studies have explored hybrid models integrating neural network components. Lithium-ion battery data inherently exhibit complex spatiotemporal characteristics that single deep learning methods may inadequately capture. In contrast, hybrid models combining convolutional and recurrent architectures can exploit complementary advantages to comprehensively utilize both temporal dependencies and multi-indicator feature information. Huang et al.^15^proposed a CNN-LSTM (Long Short-Term Memory)-TPA (Temporal Pattern Attention) model with WOA hyperparameter optimization, achieving average Mean Absolute Error (MAE) of 0.44% on NASA dataset. However, the method extracts features from multiple health factors with equal importance and uses static TPA weights after training, lacking dynamic adjustment across different aging stages. Chen et al.^16^combined fractional-order RC equivalent circuit model with an improved vision transformer, using hybrid FPSO (Fractional Particle Swarm Optimization)-CL for parameter identification and correlation-based feature selection from both measured data and model parameters. However, the method requires extensive Equivalent Circuit Model (ECM) parameter identification for each battery, increasing computational cost and limiting scalability.

In addition to architectural innovations, recent research has explored strategies addressing practical deployment constraints such as limited labeled data and cross-manufacturer generalization. Wang et al.^17^proposed a physics-informed neural network integrating empirical degradation and state-space equations, validated on 387 batteries from four manufacturers with Mean Absolute Percentage Error (MAPE) of 0.87%. However, the method requires domain expertise to model degradation dynamics and relies on short-term pre-full-charge data, which limits its applicability when full charging profiles are unavailable. Xiang et al.^18^introduced a semi-supervised biGRU-GPR approach using only 1.5%−15% labeled calibration data, extracting features from dynamic discharge curves with RMSPE of 1.91% on NASA dataset. However, the method requires periodic calibration and struggles with long-term degradation when labeled data is sparse.

Despite the progress from single-indicator to multi-indicator approaches and from simple models to sophisticated hybrid architectures, most existing data-driven methods exhibit fundamental limitations in three aspects: lack of adaptive feature weighting mechanisms that account for time-varying indicator correlations, insufficient modeling of evolving cross-variable dependencies throughout battery lifecycle, and reliance on manual hyperparameter tuning rather than intelligent optimization. These shortcomings significantly impact estimation accuracy under complex, non-stationary aging patterns.

Beyond prediction accuracy, recent research has begun exploring additional critical performance dimensions for practical BMS deployment. A notable advancement in this direction is the work by Chen et al.^23^, who developed a scalable deep recurrent structure combining LSTM and Gaussian Process Regression for battery health prognosis under fast-charging conditions. Their framework addresses the computational scalability challenge inherent in traditional GPR methods—which suffer from O(n³) complexity due to kernel matrix inversion^24^—by employing structured kernel interpolation and semi-stochastic gradient descent to achieve O(n) training complexity while maintaining high prediction accuracy. This represents a significant step toward enabling efficient large-scale battery health monitoring. Additionally, uncertainty quantification has emerged as an important consideration, with probabilistic approaches naturally providing confidence intervals through posterior distributions^25,26^. These developments highlight promising directions for enhancing SOH estimation frameworks beyond point prediction accuracy. To systematically compare existing approaches across multiple performance dimensions, Table 1 presents a comprehensive evaluation framework.

To systematically compare existing approaches with the proposed MI-SOH framework, Table 1 presents a comprehensive comparison across key methodological dimensions. As demonstrated in the table, existing methods exhibit several fundamental limitations that MI-SOH systematically addresses.

**Table 1:** Comparative analysis of MI-SOH against existing SOH Estimation methods.

Paper	Multi-Indicator Fusion	Adaptive Feature Weighting	Temporal-Spatial Modeling	Intelligent Optimization
Lin et al. (2022) [12]	✗ (single: CCCT)	N/A	✗ (Random Forest only)	✗ (manual)
Fan et al. (2023) [13]	✗ (single: 10 s relaxation voltage)	N/A	Partial (CNN spatial only)	✗ (manual)
Lin et al. (2022) [14]	✓ (7 features: V, T, IC)	✗ (equal + static RFR)	✗ (RFR ensemble only)	✗ (manual)
Huang et al. (2025) [15]	✓ (multiple HFs)	Partial (static TPA)	✓ (CNN-LSTM-TPA)	✓ (WOA)
Chen et al. (2024) [16]	✓ (measured + model HFs)	Partial (correlation-based)	Partial (ViT spatial only)	✓ (FPSO-CL)
Wang et al. (2024) [17]	✓ (statistical features)	✗ (physics-based)	Partial (state-space)	✗ (manual)
Xiang et al. (2024) [18]	✓ (3 features)	✗ (manual selection)	✓ (biGRU temporal)	✗ (manual)
MI-SOH (Proposed)	✓ (8 features: $\:{F}_{1\:}$-$\:{F}_{8\:}$)	✓ (Dual-correlation)	✓ (TCN + iTransformer)	✓ (ZOA)

Note: “✓” = fully implemented; “✗” = not implemented; Partial = incomplete (e.g., static attention, spatial-only modeling); N/A = not applicable.

In response to the challenges mentioned above, this paper proposes MI-SOH framework addresses the limitations of existing methods by incorporating multi-indicator feature weighting, temporal-adaptive learning mechanisms, and global hyperparameter optimization, providing a comprehensive solution for accurate and robust SOH estimation across diverse battery aging scenarios.

## Methodology

SOH represents the energy storage capability of an aged battery relative to a new one, mathematically defined as:

1 $$\:\begin{array}{c}SOH=\frac{{C}_{current}}{{C}_{initial}}\times\:100\%\end{array}$$

Where$\:\:{C}_{current\:}$and$\:\:{C}_{initial\:}$represent the current and initial maximum available capacity, respectively.

As lithium-ion batteries undergo charge-discharge cycles, internal resistance increases and capacity decreases due to complex electrochemical degradation mechanisms including electrode material loss, electrolyte decomposition, and lithium plating. These degradation processes create non-linear, time-varying relationships between observable parameters (voltage, current, temperature) and the underlying SOH, making accurate estimation challenging. Current methods struggle to capture these evolving interdependencies, necessitating advanced frameworks that can model the complex, multivariate nature of battery health degradation.

### MI-SOH framework overview

**Fig. 1:**
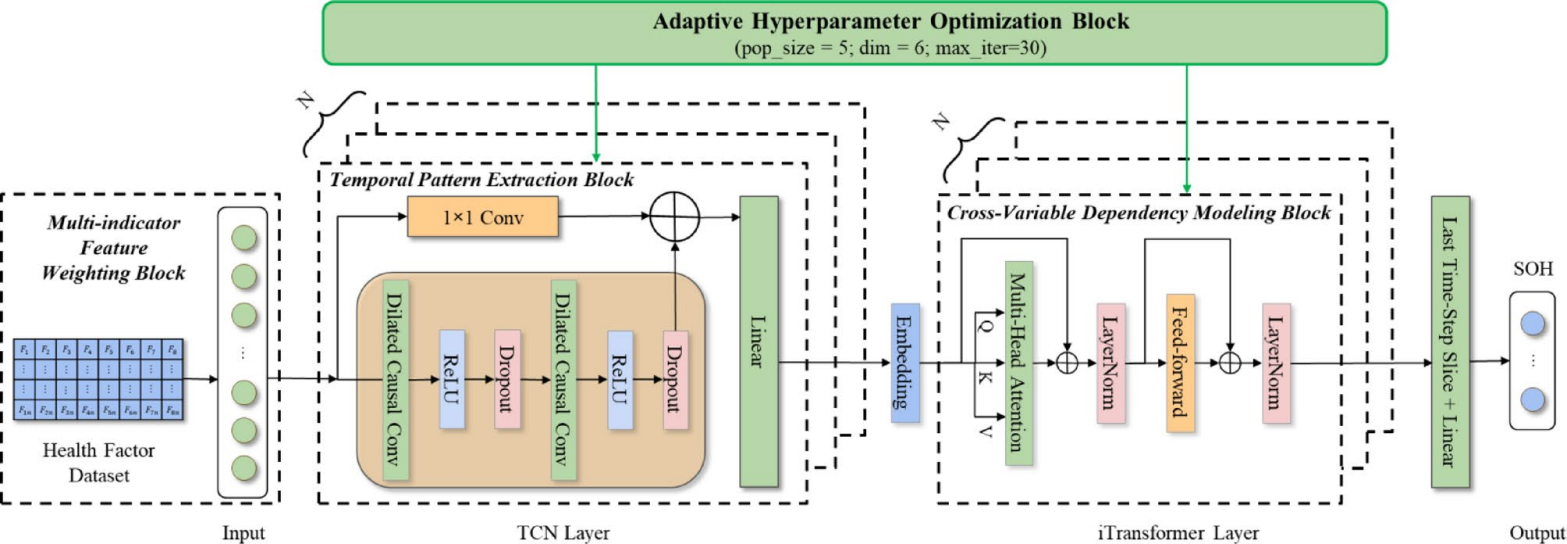
The Structure of the MI-SOH Model.

To address the limitations of existing SOH estimation methods that rely on traditional indicators, we propose MI-SOH framework, as illustrated in Fig. 1. the MI-SOH framework consists of four key components: (1) Multi-indicator Feature Weighting Block that combines Pearson correlation and grey relational analysis to assign optimal weights to health factors; (2) Temporal Pattern Extraction Block that captures short-term temporal patterns through causal dilated convolutions; (3) Cross-Variable Dependency Modeling Block that models long-range cross-variable dependencies via attention mechanisms; and (4) Adaptive Hyperparameter Optimization Block that globally optimizes all hyperparameters for adaptive performance.

These four modules are specifically designed to address critical limitations in existing SOH estimation methods. The Multi-indicator Feature Weighting Block replaces static equal-weight schemes^15,16^with adaptive dual-correlation analysis that adjusts feature importance across degradation stages. The Temporal Pattern Extraction Block employs dilated TCN instead of recurrent networks (LSTM/GRU) to enable parallel processing and multi-scale pattern capture without gradient vanishing^27,28^. The Cross-Variable Dependency Modeling Block overcomes the independent variable assumption by leveraging iTransformer’s variable-as-token paradigm to model cross-indicator interactions^29^. The Adaptive Hyperparameter Optimization Block uses ZOA’s population-based search to replace manual tuning or exhaustive grid search, achieving global optimization while reducing computational cost^30^. This integrated design captures temporal dynamics and cross-variable dependencies while maintaining efficiency for battery management applications.

The framework operates through a sequential pipeline where preprocessed multi-dimensional health factors are first weighted according to their adaptive correlation-based importance (reflecting multi-stage degradation patterns), then fed into the TCN module for local temporal feature extraction. The extracted features are subsequently processed by the iTransformer module to capture long-term dependencies and cross-variable interactions. Throughout this process, the ZOA algorithm continuously optimizes hyperparameters including kernel sizes, dilation rates, hidden dimensions, attention heads, and learning rates to ensure optimal performance across diverse battery aging scenarios. This integrated approach enables MI-SOH to effectively characterize capacity fade patterns while maintaining robustness across different battery types and operating conditions.

### Multi-indicator feature weighting block

Building upon the health factor extraction methodology described in Sect. 4.2, the multi-indicator feature weighting module addresses a fundamental challenge revealed through systematic analysis: health indicator correlations with SOH exhibit significant temporal variability throughout battery degradation stages. Our investigation demonstrates that indicator pairs undergo diverse transformations—some strengthening in relevance, others weakening, and certain correlations showing non-monotonic evolution across aging phases (detailed in Sect. “Temporal evolution analysis of indicator correlations”). Rather than employing static feature importance throughout battery lifecycle, this module implements an adaptive dual-correlation approach that dynamically adjusts feature weights based on their evolving relationships with SOH.

The weight calculation for each health factor combines Pearson correlation coefficient analysis (capturing linear dependencies) with grey relational analysis (revealing non-linear sequence-based similarities):

2 $$\:\begin{array}{c}{w}_{i}=\alpha\:\cdot\:{P}_{i}+\left(1-{\upalpha\:}\right)\cdot\:{G}_{i}\end{array}$$

Where$\:\:{P}_{i}\:$represents the Pearson correlation coefficient between the$\:\:i$-th feature and SOH, $\:{G}_{i}\:$represents the grey correlation degree, and$\:\:\:{\upalpha\:}\:\:$is the weight distribution factor determined through cross-validation optimization. This dual approach ensures that both linear correlations and complex non-linear dependencies are appropriately considered when determining feature importance.

**Fig. 2:**
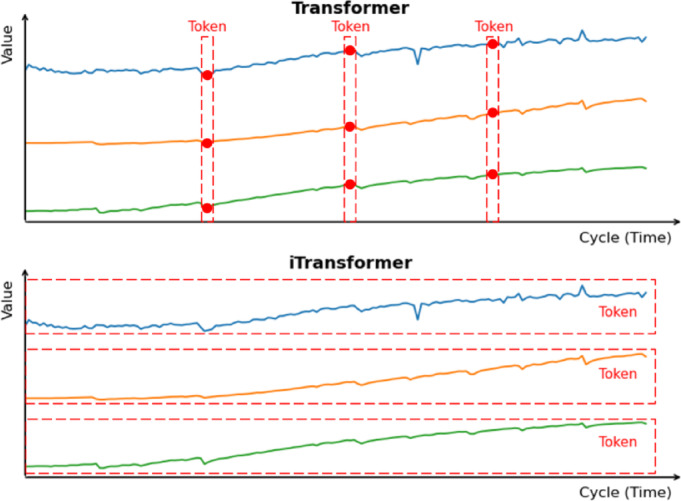
Pearson correlation coefficient heatmap (exemplified with B0005 battery).

**Fig. 3:**
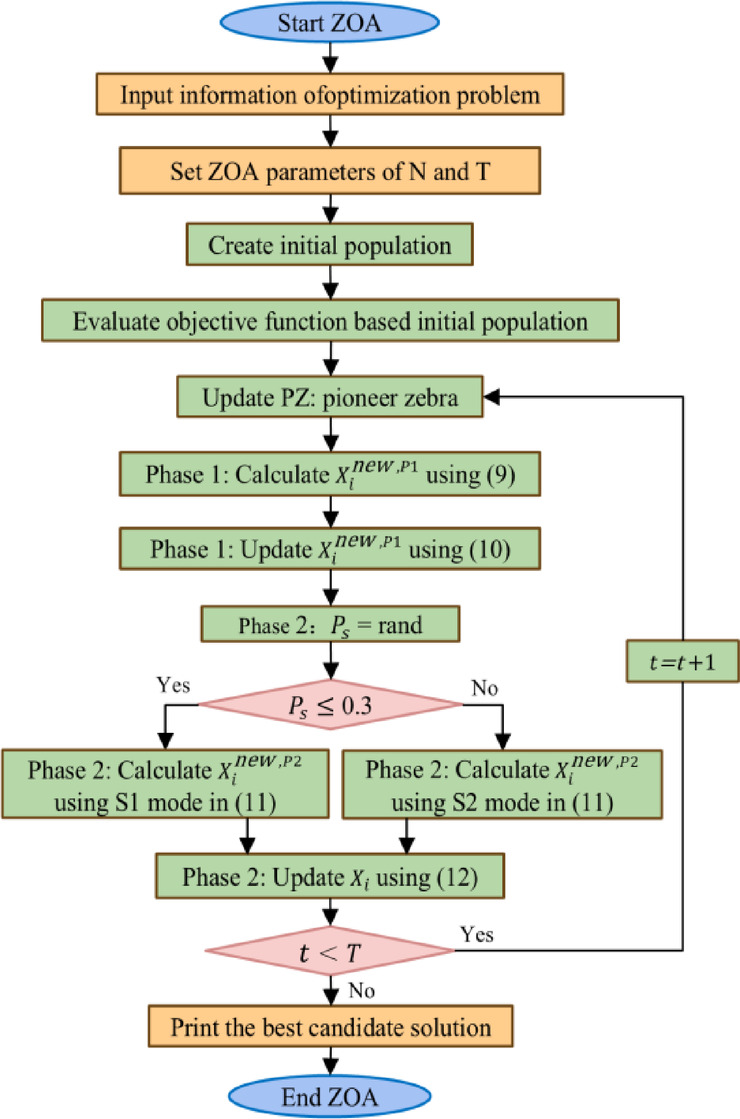
The SOH degradation curves. **(a)** NASA dataset batteries (B0005, B0006, B0007, B0018). **(b)** CALCE dataset batteries (CS2_35, CS2_36, CS2_37, CS2_38).

This dual-correlation approach is chosen for three key reasons. First, Pearson correlation effectively quantifies linear dependencies in monotonic degradation patterns—as demonstrated in Fig. 2, features $\:{F}_{3\:}$and $\:{F}_{7\:}$exhibit strong linear correlations (*r* > 0.8) with SOH. Second, grey relational analysis captures non-linear sequence-based similarities critical for handling non-monotonic behaviors such as capacity recovery phenomena (Sect. “Data preprocessing”, Fig. 3). The weighted combination (α = 0.5) balances contributions from both linear and non-linear dependencies. Third, Table 3 validates this synergy: indicators with high Pearson coefficients ($\:{F}_{3}$, $\:{F}_{7}$) consistently show strong grey relational degrees (> 0.77), confirming robust ranking across metrics. Alternative correlation methods present specific limitations for battery degradation analysis: mutual information suffers from high computational cost and sensitivity to discretization parameters^31,32^; Spearman and Kendall rank-based correlations quantify only ordinal relationships without preserving information about magnitude changes critical for degradation quantification^33^; distance correlation provides a single aggregated metric without distinguishing linear versus non-linear components^34^. The Pearson-Grey dual framework addresses these limitations while maintaining computational efficiency and interpretability for battery management applications^35,36^.

The weighting mechanism is computed on data encompassing multiple degradation stages during training, enabling the model to learn patterns of time-varying feature importance. For instance, when the correlation between constant current charging duration ($\:{F}_{3}$) and IC curve peak ($\:{F}_{7}$) weakens from 0.803 to 0.255 across degradation stages (Sect. “Temporal evolution analysis of indicator correlations”), the framework automatically reduces reliance on decoupling indicator pairs while maintaining focus on consistently informative features.

The resulting weighted feature matrix is constructed as:

3 $$\:\begin{array}{c}{X}^{\left(t\right)}=\left[{w}_{1}^{\left(t\right)}\cdot\:{F}_{1},{w}_{2}^{\left(t\right)}\cdot\:{F}_{2},\dots\:,{w}_{n}^{\left(t\right)}\cdot\:{F}_{n}\right]\end{array}$$

By incorporating this dual-correlation weighting strategy, the MI-SOH framework maintains robust estimation accuracy across diverse battery aging scenarios.

### Temporal pattern extraction block

The weighted multi-indicator features are subsequently processed by the Dilated-Causal TCN module, which serves as the temporal feature extraction backbone of the MI-SOH framework. This module addresses the inherent limitations of traditional recurrent networks in capturing long-term dependencies while maintaining computational efficiency through parallel processing capabilities essential for battery health monitoring applications^37,38^.

The TCN architecture employs causal dilated convolutions to ensure temporal causality—preventing future information leakage—while exponentially expanding the receptive field to capture temporal patterns at multiple scales^39^. The dilated causal convolution operation is mathematically defined as:

4 $$\:\begin{array}{c}F\left(s\right)=\left(x{\mathrm{*}}_{d}f\right)\left(s\right)={\sum\:}_{i=0}^{n-1}f\left(i\right)\cdot\:{x}_{s-d\cdot\:i}\end{array}$$

Where $\:s$ represents the input time series, $\:*$ denotes the convolution, $\:i$ signifies the number of filters, $\:d$ represents the dilation factor, and $\:n$ denotes the filter size.

The hierarchical structure incorporates multiple TCN layers with exponentially increasing dilation factors (1, 2, 4, 8), enabling the module to capture temporal patterns ranging from short-term fluctuations in individual charge-discharge cycles to longer-term degradation trends spanning multiple cycles. Residual connections and dropout regularization are strategically incorporated to prevent gradient vanishing problems and overfitting, ensuring stable training performance across extended battery aging sequences. This architectural design allows the TCN module to effectively extract local temporal degradation motifs from the weighted health indicators, providing rich feature representations that capture both immediate and progressive capacity fade patterns.

### Cross-Variable dependency modeling block

**Fig. 4:**
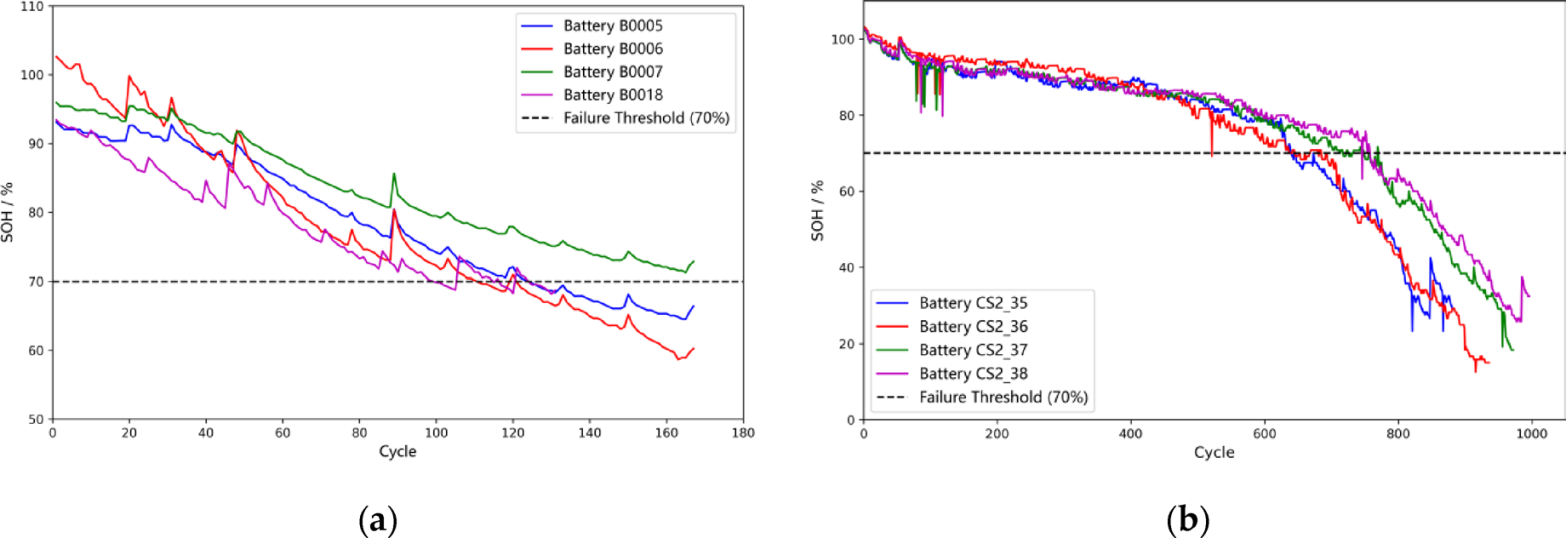
Comparison of tokenization strategies: conventional Transformer treats each time step as a token, while iTransformer treats each health indicator (exemplified with $\:{F}_{1}-{F}_{3}$) as a token.

While the Temporal pattern extraction module excels at capturing temporal dependencies within individual health indicators, accurate battery SOH estimation requires understanding the complex interactions and dependencies between different health indicators over extended time periods. The Inverted Transformer (iTransformer) module addresses this critical need by modeling cross-variable dependencies through an innovative attention mechanism that treats each health indicator as an independent token rather than processing temporal sequences along the time dimension^40,41^. The comparison method for embedding Transformer and iTransformer is shown in Fig. 4.

In this framework, a “token” represents the complete temporal sequence of a single health indicator across all time steps, contrasting with conventional Transformers where each time step serves as a token^29^. This variable-as-token paradigm proves particularly effective for battery degradation analysis because cross-variable dependencies (how voltage, current, and temperature indicators interact) are more informative for SOH estimation than fine-grained temporal patterns within individual variables^42^. The framework employs eight health indicators as tokens ($\:{F}_{1\:}$-$\:{F}_{8\:}$, detailed in Sect. “Health factor extraction”), selected through comprehensive correlation analysis to collectively capture voltage, current, temperature, and electrochemical degradation characteristics essential for SOH estimation^43^. This tokenization reduces computational complexity from O(L²) to O(N²), where *N* = 8 health indicators and L represents sequence length.

Unlike conventional Transformers that compute attention along the temporal axis, the iTransformer operates on the variable dimension, enabling efficient modeling of multivariate correlations^44^. Given a sequence $\:\left\{{\boldsymbol{X}}_{1},{\boldsymbol{X}}_{2},\cdots\:,{\boldsymbol{X}}_{m}\right\}\in\:{R}^{m\times\:l}$ with $\:m$ time variables and a lag length of $\:l$ to get the results $\:\left\{{\widehat{\boldsymbol{Y}}}_{1},{\widehat{\boldsymbol{Y}}}_{2},\cdots\:,{\widehat{\boldsymbol{Y}}}_{m}\right\}\in\:{R}^{m\times\:p}$ with desired forecast length $\:p$, the iTransformer embeds each individual variable separately.

5 $$\:\begin{array}{c}{h}_{i}^{0}=Embedding\left({X}_{i}\right),i=\mathrm{1,2},\cdots\:,m,\end{array}$$

6 $$\:\begin{array}{c}\widehat{{h}_{i}^{l}}=LN\left({h}_{i}^{l}+\mathrm{S}\mathrm{A}\left({h}_{i}^{l}\right)\right),l=\mathrm{0,1},\cdots\:,L-1,\end{array}$$

7 $$\:\begin{array}{c}{h}_{i}^{l+1}=\mathrm{L}\mathrm{N}\left(\mathrm{F}\mathrm{F}\mathrm{N}\left(\widehat{{h}_{i}^{l}}\right)+\widehat{{h}_{i}^{l}}\right),l=\mathrm{0,1},\cdots\:,L-1,\end{array}$$

8 $$\:\begin{array}{c}\widehat{{Y}_{i}}=Projection\left({h}_{i}^{L}\right),\end{array}$$

Where $\:\mathrm{E}\mathrm{m}\mathrm{b}\mathrm{e}\mathrm{d}\mathrm{d}\mathrm{i}\mathrm{n}\mathrm{g}\left(\cdot\:\right)$ embeds the sequence length $\:l$ into $\:{d}_{h}$, while the $\:\mathrm{P}\mathrm{r}\mathrm{o}\mathrm{j}\mathrm{e}\mathrm{c}\mathrm{t}\mathrm{i}\mathrm{o}\mathrm{n}\left(\cdot\:\right)$ projects $\:{\boldsymbol{h}}_{\boldsymbol{i}}^{\boldsymbol{L}}\in\:{R}^{1\times\:{d}_{h}}$ to the desired forecast $\:{\widehat{Y}}_{i}\in\:{R}^{1\times\:p}$ and $\:L$ is the layer of the iTransformer. Both operations are implemented through multi-layer perceptron (MLP). $\:\mathrm{LN}\left(\cdot\:\right)$ is the Layer Normalization operation and $\:\mathrm{F}\mathrm{F}\mathrm{N}\left(\cdot\:\right)$ denotes the feed forward network. This architecture reduces computational complexity from O(L²) to O(N²) when the number of variables N is substantially smaller than sequence length L.

The iTransformer module effectively captures how different health indicators—voltage characteristics, current patterns, temperature variations, and electrochemical properties—influence each other throughout the battery’s operational lifecycle. This cross-variable modeling capability proves crucial for accurate SOH estimation, as battery degradation emerges from the complex interplay of multiple interdependent physical and chemical processes that cannot be adequately captured through individual indicator analysis.

### Adaptive hyperparameter optimization block

To achieve optimal performance across diverse battery types and aging scenarios, the MI-SOH framework incorporates an adaptive hyperparameter optimization block for comprehensive global hyperparameter optimization. Traditional approaches such as grid search and manual parameter tuning prove inadequate for navigating the complex, high-dimensional parameter space of the integrated TCN-iTransformer architecture, necessitating a more sophisticated optimization strategy.

The ZOA algorithm simulates the collective foraging and defense behaviors observed in zebra populations, providing robust global search capabilities for hyperparameter optimization tasks^30^. The algorithm maintains a population of candidate solutions, with each zebra representing a complete hyperparameter configuration encompassing TCN kernel sizes, dilation rates, hidden dimensions, iTransformer attention heads, learning rates, and other critical architectural parameters.

During the foraging phase, population members are updated by moving toward the best-performing solution (designated as the pioneer zebra):

9 $$\:\begin{array}{c}{x}_{i,j}^{\mathrm{new\:},P1}={x}_{i,j}+r\cdot\:\left(P{Z}_{j}-I\cdot\:{x}_{i,j}\right)\end{array}$$

10 $$\:\begin{array}{c}{X}_{i}=\left\{\begin{array}{c}{X}_{i}^{\mathrm{new\:},P1},\:\:{F}_{i}^{\mathrm{new\:},P1}<{F}_{i}\\\:{X}_{i},\:\:else\end{array}\right.\end{array}$$

Where$\:\:{X}_{i}^{\mathrm{new\:},P1\:}$represents the updated state of the$\:\:i$-th zebra in the first phase, $\:{x}_{i,j}^{\mathrm{new\:},P1\:}$is its value in the $\:j$-th dimension, and $\:{F}_{i}^{\mathrm{new\:},P1\:}$is its objective function value. $\:PZ\:$represents the pioneer zebra, and $\:P{Z}_{j\:}$is its value in the$\:\:j$-th dimension. $\:r\:$is a random number within the interval [0, 1], and $\:I=round(1+rand)$, where $\:rand\:$is a random number within the interval [0, 1]. Therefore, $\:I\in\:\left\{\mathrm{1,2}\right\}$. If the parameter $\:I=2$, the population movement will be significantly larger.

During the defense phase, zebras adapt to environmental threats through two distinct behavioral strategies depending on predator characteristics^45^:

11 $$\:\begin{array}{c}{x}_{i,j}^{\mathrm{new\:},P2}=\left\{\begin{array}{c}{S1:x}_{i,j}+R\cdot\:\left(2r-1\right)\cdot\:\left(1-\frac{t}{T}\right){\cdot\:x}_{i,j},{P}_{s}\le\:0.3\\\:{S2:x}_{i,j}+r\cdot\:(A{Z}_{j}-I{\cdot\:x}_{i,j}),\:else\end{array}\right.\end{array}$$

During the position update process for both foraging and defense phases, new solutions are accepted only when they demonstrate improved performance (lower objective function values for minimization problems). The selection criterion is expressed as:

12 $$\:\begin{array}{c}{X}_{i}=\left\{\begin{array}{c}{X}_{i}^{\mathrm{new\:},P2},\:\:{F}_{i}^{\mathrm{new\:},P2}<{F}_{i}\\\:{X}_{i},\:\:else\end{array}\right.\end{array}$$

Where$\:\:{X}_{i}^{\mathrm{new\:},P2}\:$represents the updated position of the $\:i$-th zebra in the second phase, $\:{x}_{i,j}^{\mathrm{new\:},P2}\:$represents its value in the$\:\:j$-th dimension, and$\:\:{F}_{i}^{\mathrm{new\:},P2\:}$is its objective function value. $\:t\:$ represents the iteration number, $\:T\:$is the maximum number of iterations, $\:R\:$is a constant equal to 0.01, and $\:{P}_{s\:}$is the probability of choosing one of two randomly generated strategies within the interval [0, 1]. $\:AZ\:$represents the zebra under attack, and $\:A{Z}_{j}\:$is its value in the $\:j$-th dimension.

**Fig. 5:**
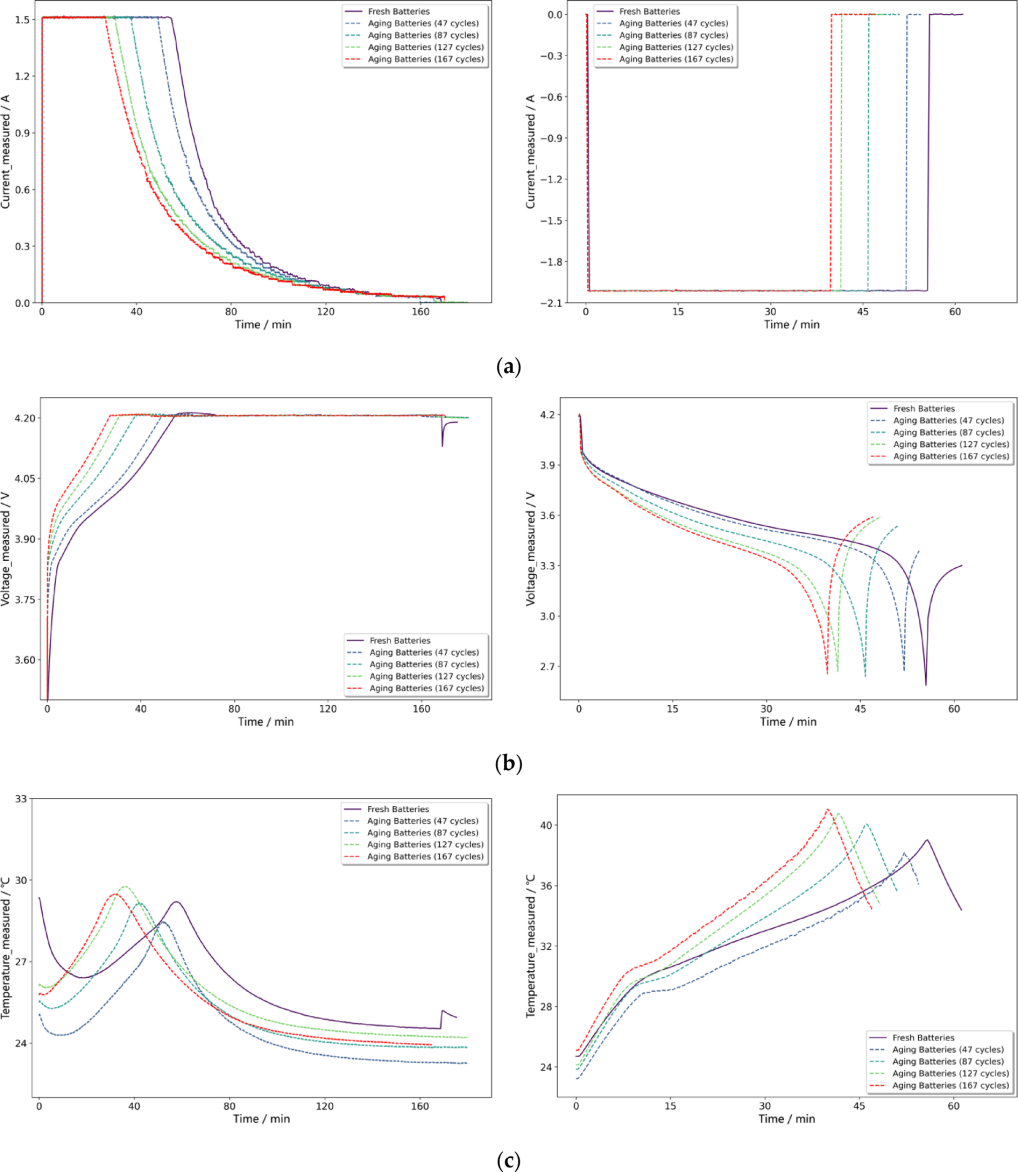
ZOA Flowchart.

During each iteration of ZOA, the population members are updated based on foraging and defense strategies. The continuous update process of the algorithm population follows (9) to (12) until the entire algorithm execution is complete. The best candidate solution is continuously updated and retained throughout the consecutive iterations. The specific implementation process is illustrated in Fig. 5.

This dual-phase optimization process enables the ZOA module to efficiently explore the hyperparameter space while avoiding local optima, ensuring that the MI-SOH framework adapts optimally to different battery degradation patterns and operating conditions. The integration of ZOA optimization eliminates the dependency on manual hyperparameter tuning and enables the framework to automatically configure itself for maximum SOH estimation accuracy across diverse battery chemistries and usage scenarios.

## Experiment

### Dataset

To comprehensively validate the proposed MI-SOH framework, this study employs two publicly available lithium-ion battery datasets with distinct chemistries and form factors: the NASA Glenn Research Center dataset featuring NCA cylindrical 18,650 cells, and the CALCE battery dataset from the University of Maryland featuring LCO prismatic CS2 cells. Each battery underwent accelerated aging through systematic charge-discharge cycling protocols. The failure threshold is set when the capacity degrades to 70% of the rated capacity, indicating the battery’s failure^46^. The detailed experimental information is shown in Table 2.

**Table 2:** The battery specifications of the NASA and CALCE datasets.

Battery category	NASA				CALCE			
	B0005	B0006	B0007	B0018	CS2_35	CS2_36	CS2_37	CS2_38
Rated capacity (Ah)	2.0	2.0	2.0	2.0	1.1	1.1	1.1	1.1
Charging current (A)	1.5	1.5	1.5	1.5	0.55	0.55	0.55	0.55
Discharge current (A)	2.0	2.0	2.0	2.0	1.1	1.1	1.1	1.1
Cut-off voltage (V)	2.7	2.5	2.2	2.5	2.7	2.7	2.7	2.7
Ambient temperature (℃)	24.0	24.0	24.0	24.0	25.0	25.0	25.0	25.0
Total cycles	168	168	168	132	882	936	972	996

The SOH degradation curves of the lithium-ion batteries are shown in Fig. 3. Each cycle represents one aging experiment, and as the battery ages, its internal resistance increases and capacity decreases. The black dashed line represents the failure threshold^27^. NASA batteries (Fig. 3a) exhibit diverse aging patterns over short lifecycles (132–168 cycles), with varying degradation rates across B0005-B0018. CALCE batteries (Fig. 3b) feature extended lifecycles (882–996 cycles) with multi-stage degradation characteristics, demonstrating stable early-stage performance followed by accelerated late-stage fade. The degradation trajectories exhibit non-monotonic behavior with occasional capacity recovery phenomena due to rest intervals and measurement uncertainties, underscoring the complexity of accurate SOH estimation.

**Fig. 6:**
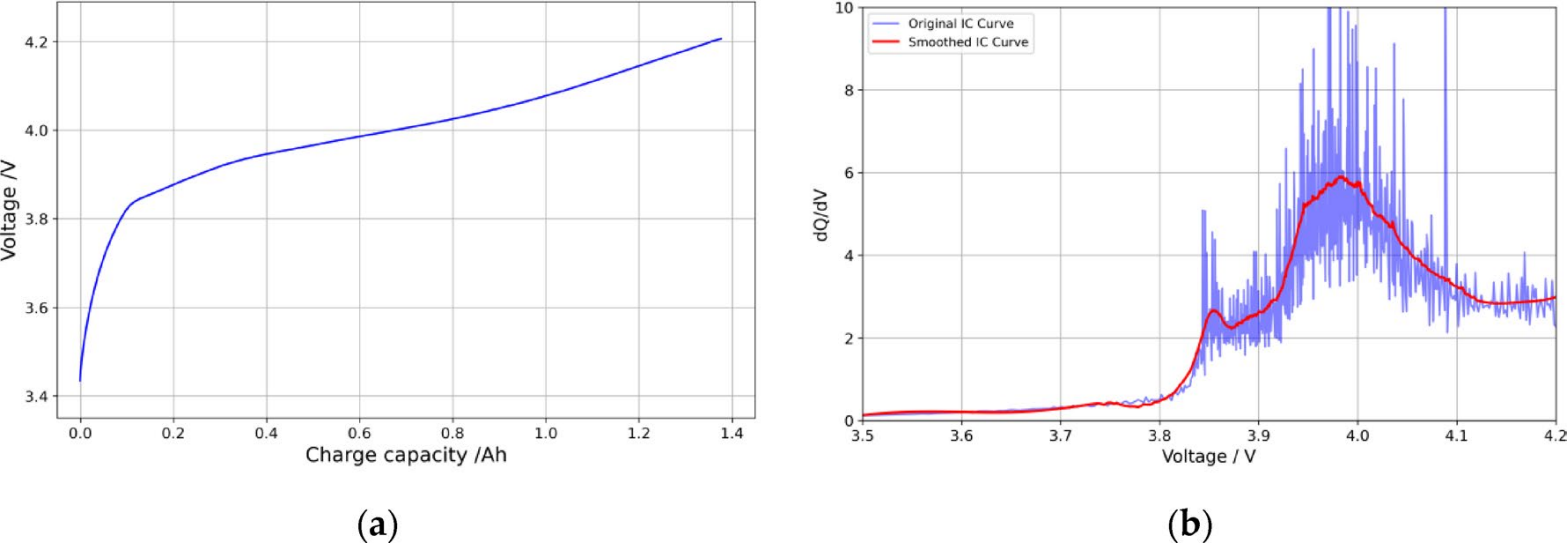
The charge and discharge curve information of the B0005 lithium-ion battery. **(a)** The charge/discharge current–time curves. **(b)** The charge/discharge voltage–time curves. **(c)** The charge/discharge temperature–time curves.

Due to the uncertainty in the discharge process of lithium-ion batteries, such as user habits and environmental temperature, the collected discharge data may not be objective. The health state of the battery is influenced by various factors, making it difficult to use discharge data for SOH estimation. During the charging process, however, the battery is generally not in use and does not output energy, which reduces interference, making the data relatively objective. Therefore, constant current and constant voltage charging processes are commonly used for data analysis when measuring battery health status^47^. Taking the B0005 battery as an example, Fig. 6 shows the charge-discharge voltage, current, and temperature variation curves for five cycles at 2, 47, 87, 127, and 167 cycles. From Figs. 6(a) to (c), it can be observed that as the number of cycles increases, compared to the initial cycle, the duration of the constant current charging stage shortens, the voltage reaches 4.2 V more quickly, the temperature peak appears earlier, and the discharge time to the cutoff voltage is reduced. These analyses indicate that the battery’s current, voltage, and temperature influence its SOH.

### Data preprocessing

#### Health factor extraction

In this study, several health factors related to SOH are selected as the health factor dataset for both NASA and CALCE datasets. These include the duration of constant voltage charging$\:{\:F}_{1}$, the voltage change rate$\:{\:F}_{2}$, the duration of constant current charging$\:\:{F}_{3}$, the current change rate$\:\:{F}_{4}$, the average temperature on the battery surface during charging$\:\:{F}_{5}$, the maximum temperature on the battery surface during charging$\:{\:F}_{6}$, and the peak$\:{\:F}_{7\:}$and corresponding voltage$\:{\:F}_{8}\:$of the IC curve.

As research indicates, as the battery gradually ages, the internal lithium-ion concentration decreases, and the active materials are gradually consumed. When the same battery charges the same amount of energy, significant differences in the charging voltage change trend may occur. Therefore, based on the charging voltage data of lithium batteries, the health factors$\:\:{F}_{1}$、$\:{F}_{2\:}$are extracted to describe the aging characteristics of batteries.

The duration of constant voltage charging ($\:{F}_{1}$) during the battery charging stage is defined as the time interval between when the battery’s charging voltage reaches the set constant voltage value (e.g., 4.2 V) and the end of charging, as shown in (13):

13 $$\:\begin{array}{c}{F}_{1}={t}_{end}-{t}_{const}\end{array}$$

Where$\:{\:t}_{end\:}$represents the end of the charging stage, and$\:\:{t}_{const}\:$is the time when the charging voltage first reaches the constant voltage value$\:({V}_{set}=4.2V\pm\:\varDelta\:V)$, with$\:\:\varDelta\:V\:$represents a small voltage margin.

The voltage change rate ($\:{F}_{2}$) is defined by fitting the data points of the battery’s constant current charging voltage within the range of 3.8–4.2 V, with the calculation process shown in (14):

14 $$\:\begin{array}{c}{F}_{2}=\frac{{\sum\:}_{j=1}^{n}\left({t}_{j}-\stackrel{-}{t}\right){V}_{i}}{{\sum\:}_{j=1}^{n}{\left({t}_{j}-\stackrel{-}{t}\right)}^{2}}\end{array}$$

Where$\:\:{V}_{i\:\:}$is the charging voltage value of the battery at the time point$\:\:{t}_{j\:}$; $\:\stackrel{-}{t}\:$is the average value of the charging time; $\:n\:$indicates the number of data points in the selected interval.

The accumulation of charge primarily depends on the constant current charging stage. A reduction in the duration of the constant current charging stage is associated with a decrease in the current charging capacity, an increase in the battery polarization, and a reduction in the lithium-ion charge in the positive electrode, all of which affect the SOH. Therefore, based on the charging current data of lithium batteries, the health factors$\:\:{F}_{3}$、$\:{F}_{4\:}$are extracted to describe the aging characteristics of batteries.

The duration of constant current charging ($\:{F}_{3}$) during the battery charging stage refers to the total time when the charging current remains at 1.5 A, as shown in (15):

15 $$\:\begin{array}{c}{F}_{3}={t}_{const}-{t}_{start}\end{array}$$

Where$\:{\:t}_{const\:}$is the time when the charging current first deviates from the set constant current value$\:{(I}_{set}=1.5A\pm\:\varDelta\:I)$, and$\:{\:t}_{start\:}$represents the start of the charging stage, with$\:\:\varDelta\:I\:$represents a small current margin.

The current change rate ($\:{F}_{4}$) is defined by fitting the data points of the battery’s constant voltage charging current within the range of 0.1–1.4 A, with the calculation process shown in (16):

16 $$\:\begin{array}{c}{F}_{4}=\frac{{\sum\:}_{j=1}^{n}\left({t}_{j}-\stackrel{-}{t}\right){I}_{i}}{{\sum\:}_{j=1}^{n}{\left({t}_{j}-\stackrel{-}{t}\right)}^{2}}\end{array}$$

Where$\:{\:I}_{i\:}$is the charging current value of the battery at the time point$\:\:{t}_{j\:}$; $\:\stackrel{-}{t}\:$is the average value of the charging time;$\:\:n\:$indicates the number of data points in the selected interval.

During the charging and discharging processes of the battery, external environmental temperature, switching charging and discharging strategies, and the internal chemical reactions all influence the migration rate of lithium ions and the conductivity of the electrolyte. As the external temperature decreases, the internal resistance of the battery increases, the electrochemical reaction rate slows down, and the discharge capacity decreases, ultimately affecting the battery’s power and energy output. Under different aging conditions, the internal resistance of the battery gradually increases, and the chemical reactions inside the battery cause the surface temperature to rise. Therefore, based on the charging temperature data of lithium batteries, the health factors$\:\:{F}_{5}$、$\:{F}_{6\:}$are extracted to describe the aging characteristics of the battery.

The average temperature ($\:{F}_{5}$) and maximum temperature ($\:{F}_{6\:}$) on the battery surface during the charging stage are defined by (17) and (18), respectively:

17 $$\:\begin{array}{c}{F}_{5}=\frac{1}{n}{\sum\:}_{j=1}^{n}{T}_{j}\end{array}$$

18 $$\:\begin{array}{c}{F}_{6}=\underset{j\in\:\left[1,n\right]}{\mathrm{ma}\mathrm{x}}{T}_{j}\end{array}$$

Where$\:{\:T}_{j\:}$is the battery surface temperature at time$\:\:j$, and$\:\:n\:$is the total number of time points during the charging stage.

**Fig. 7:**
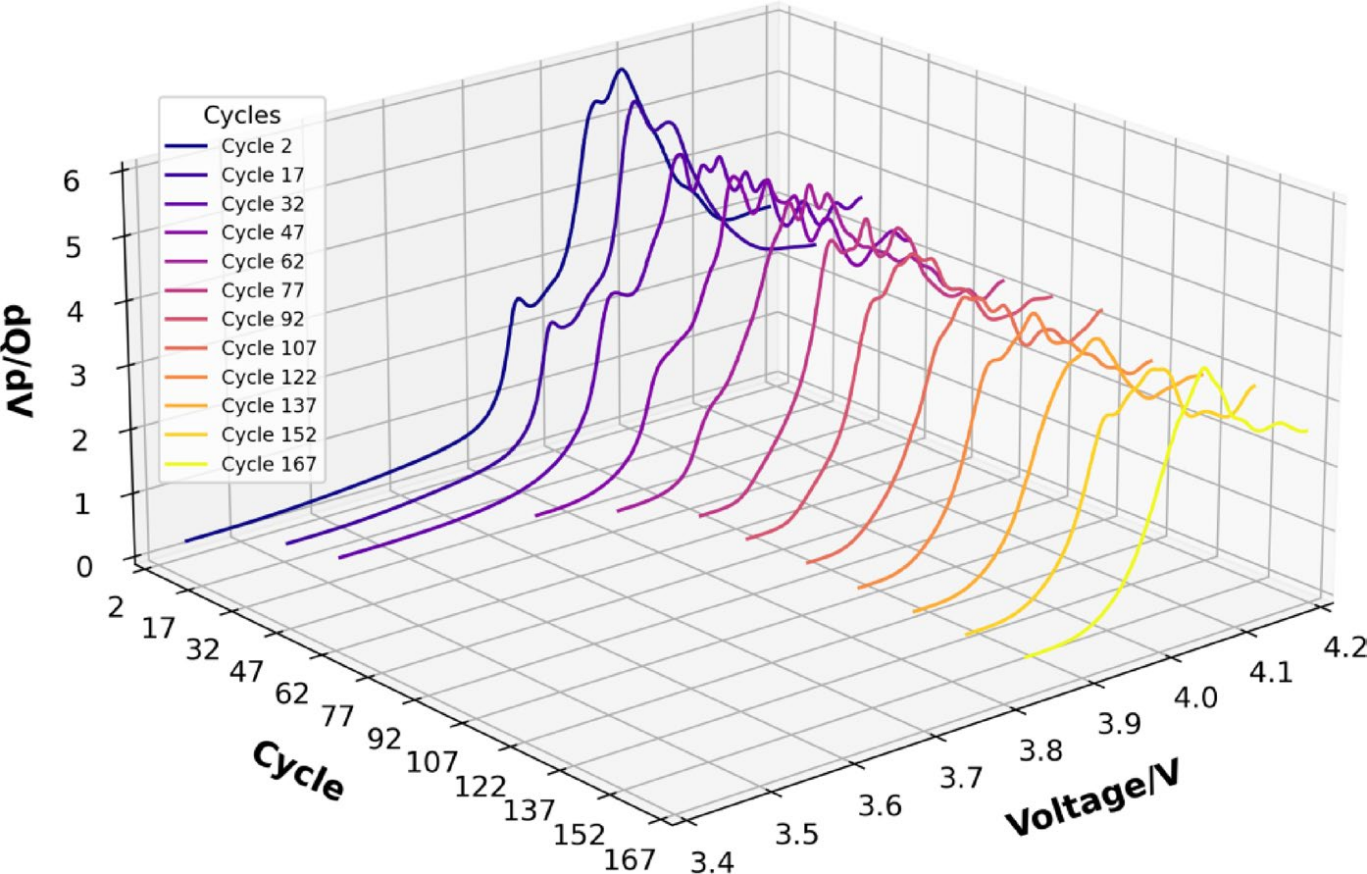
The voltage and capacity variation rate curves of lithium-ion batteries during the charging process. **(a)** The capacity-voltage curve. **(b)** The IC curves before and after filtering.

Incremental Capacity Analysis (ICA) is an important method for analyzing the material properties and degradation mechanisms of lithium-ion batteries. The core of ICA is the battery’s incremental capacity (IC) curve, which can effectively infer the chemical changes inside the lithium-ion battery and the corresponding characteristics and patterns of SOH changes^48^. As shown in Fig. 7(a), for a fresh battery, during the beginning and end of the charging process, the voltage rises rapidly. However, during the middle part of the charging process, the voltage increases slowly. In fact, the majority of the capacity has already been charged during the slow voltage rise phase. ICA refers to processing the raw charging data, calculating the$\:\:dQ/dV\:$data during the charging or discharging process, and obtaining the$\:\:dQ/dV-V\:$curve.

The basic idea is to replace$\:\:dV\:$with a fixed voltage interval$\:\:\delta\:V$, calculate the change in battery charge$\:\:\delta\:Q\:$within each$\:\:\delta\:V\:$voltage change interval, and replace$\:\:dQ\:$with$\:\:\delta\:Q$. This allows$\:\:\delta\:Q/\delta\:V\:$to replace$\:\:dQ/dV\:$, where the voltage intervals are divided by$\:\:\delta\:V$. As$\:\:\delta\:V\to\:0$, it can be written as:

19 $$\:\begin{array}{c}\frac{dQ}{dV}\approx\:\frac{\delta\:Q}{\delta\:V}\end{array}$$

In the constant current charging mode, by differentiating the charging capacity with respect to the terminal voltage, the slowly changing voltage plateau can be transformed into a distinct peak on the IC curve. The relationship between charging capacity and voltage can be described as$\:\:Q=\int\:Idt$, and$\:\:V=f\left(Q\right)$,$\:\:Q={f}^{-1}\left(V\right)$, which leads to the following:

20 $$\:\begin{array}{c}{\left({f}^{-1}\right)}^{{\prime\:}}=\frac{\mathrm{d}Q}{\mathrm{d}V}=\frac{I\mathrm{d}t}{\mathrm{d}V}=I\frac{\mathrm{d}t}{\mathrm{d}V}\end{array}$$

**Fig. 8:**
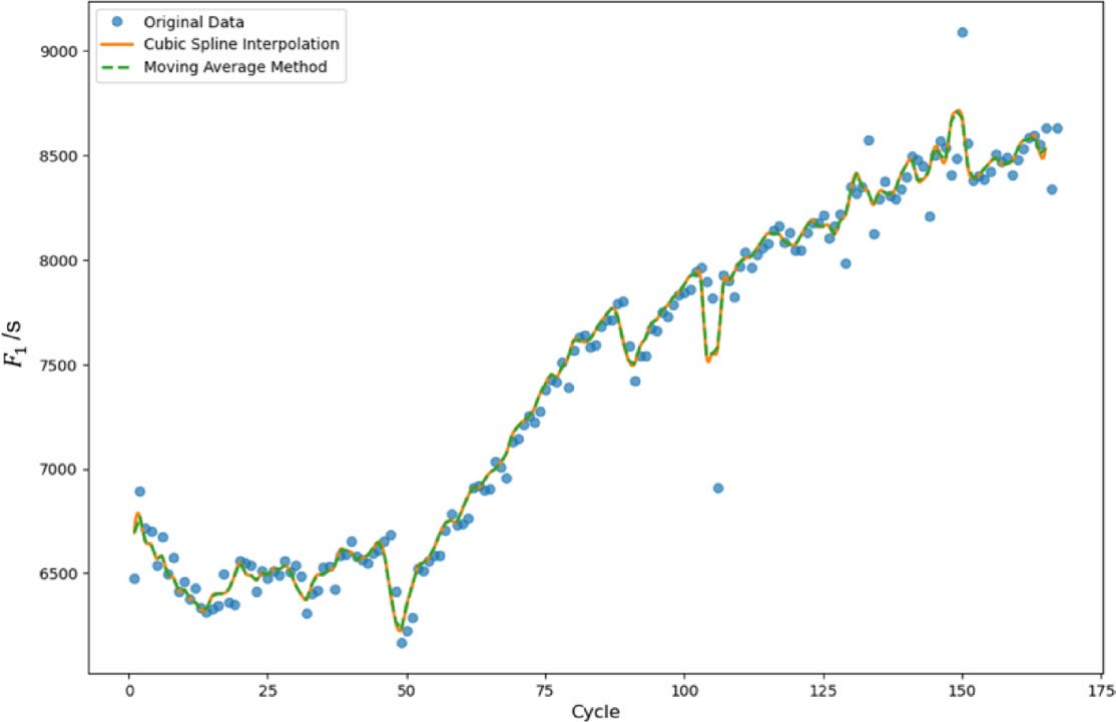
The feature extraction based on IC curves.

The battery IC curve can be obtained from Eq. (20). However, sampling errors or noise interference cause the original IC curve to be non-smooth, hindering the extraction of implicit features. To solve this problem, many filters are used to suppress noise and obtain a smooth IC curve^49^. To reduce noise, this paper applies the Savitzky-Golay filter to smooth the IC curve. Compared to other filtering methods, the Savitzky-Golay filter retains more high-frequency components (such as rapid voltage fluctuations) while effectively removing noise. This is particularly suitable for the IC curve, especially when removing periodic noise and avoiding over-smoothing. The filtered IC curves are shown in Fig. 7(b). The IC curve for different aging states is shown in Fig. 8. The results demonstrate that with increasing cycles, the peak gradually shifts toward higher voltages, and the IC peak decreases, following a monotonically decreasing trend with battery capacity. This change indicates that the IC value at the peak and the corresponding voltage can effectively characterize battery degradation. Therefore, based on the curve data of lithium battery charging IC, the health factors$\:\:{F}_{7}$、$\:{F}_{8\:}$are extracted to describe the aging characteristics of the battery.

$\:{F}_{7}\:$and$\:\:{F}_{8\:}$represent the peaks of the IC curve and their corresponding voltages after being filtered by the Savitzky-Golay filter, as shown in (21) and (22):

21 $$\:\begin{array}{c}{F}_{7}=\underset{i\in\:\left[1,m\right]}{\mathrm{max}}{IC}_{i}\end{array}$$

22 $$\:\begin{array}{c}{F}_{8}={V}_{{i}^{\mathrm{*}}}\end{array}$$

Where$\:{\:IC}_{i\:}$represents the value of the processed IC curve at the data point$\:\:i$; $\:m\:$is the total number of data points in the IC curve; $\:{i}^{\mathrm{*}}\:$is the index of the data point corresponding to the peak value$\:{\:IC}_{i}\:$; and $\:{V}_{{i}^{\mathrm{*}}\:}$represents the voltage value at the peak point$\:{\:i}^{\mathrm{*}}$.

**Fig. 9:**
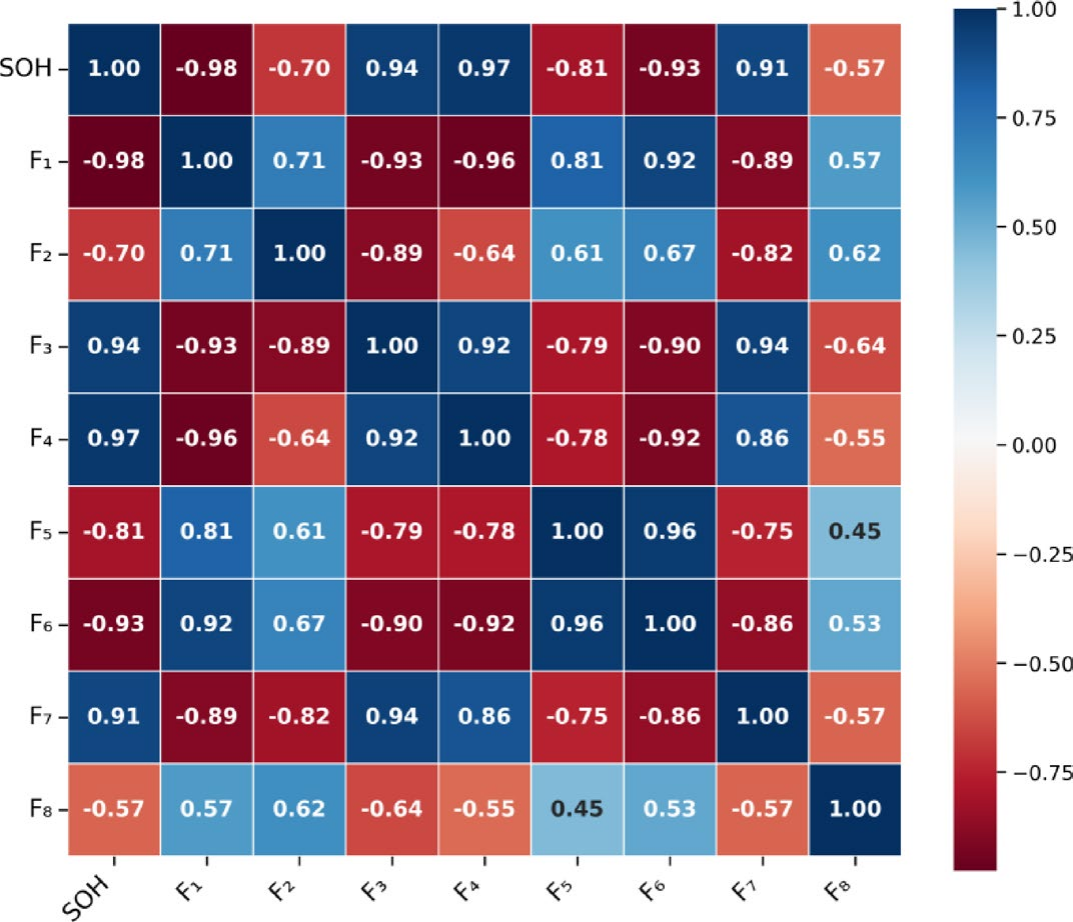
Comparison of data preprocessing effects: raw data, smooth data, and interpolation data.

Health factor data for lithium-ion batteries often contains noise and fluctuations during actual testing, which can interfere with subsequent model training. To improve data quality, this paper employs a preprocessing approach combining moving average smoothing and cubic spline interpolation methods. The moving average method is first applied to mitigate noise in the charging data by calculating the mean within a specified window, effectively smoothing short-term fluctuations while preserving long-term degradation trends and reducing random noise interference. However, the smoothed data may still suffer from sparse distribution or irregular sampling intervals. To address these issues, cubic spline interpolation is subsequently applied for further refinement. This curve fitting technique generates continuous, smooth function curves that maintain the physical significance of the data while eliminating residual fluctuations that could compromise model training effectiveness. Figure 9 demonstrates the complete preprocessing workflow using health factor $\:{F}_{1}\:$as an illustrative example.

#### Feature correlation analysis

Although the extracted health factors reflect the degradation trend of the battery’s SOH to some extent, the variation trends of each feature are not consistent. For data-driven models, the quality of the input dataset directly affects the accuracy of the model’s output. Therefore, a method is required to evaluate the relationship between different features and the actual SOH of the battery, and then assign different weights to these features as inputs for the estimation model. In this study, a combined approach using Pearson correlation coefficient and grey relational analysis is adopted to comprehensively analyze the correlation between health factors and the actual SOH of the battery. By leveraging the complementary nature of these two methods, we can more thoroughly and accurately assess the effectiveness of each feature and provide reliable data support for subsequent model construction.

The Pearson correlation coefficient is a linear correlation analysis method that quantitatively evaluates the linear relationship between two variables^50^. It is suitable for revealing strong linear correlations between features and SOH, as shown in (23). Taking the B0005 battery as an example, the Pearson correlation coefficient heatmap is shown in Fig. 2.

23 $$\:\begin{array}{c}r=\frac{{\sum\:}_{i=1}^{n}\left({X}_{i}-\stackrel{-}{X}\right)\left({Y}_{i}-\stackrel{-}{Y}\right)}{\sqrt{{\sum\:}_{i=1}^{n}{\left({X}_{i}-\stackrel{-}{X}\right)}^{2}}\sqrt{{\sum\:}_{i=1}^{n}{\left({Y}_{i}-\stackrel{-}{Y}\right)}^{2}}}\end{array}$$

Where$\:{\:X}_{i\:\:}$and$\:\:{Y}_{i\:\:}$are the $\:i\:$-th observations of the two variables$\:\:X\:$and $\:Y$, respectively (in this paper, $\:X\:$represents the actual SOH values of the battery cycles, and$\:\:Y\:$represents the extracted feature data). $\:\:\stackrel{-}{X\:}$and$\:\:\stackrel{-}{Y}\:$are the sample means for$\:\:X\:$and $\:Y$, respectively. $\:n\:$is the number of samples.

From the correlation heatmap, it can be seen that health factors ($\:{F}_{1\:}$-$\:{F}_{8\:})\:$exhibit strong correlations with the battery’s SOH, indicating that these features can reflect the changes in SOH to some extent. However, the Pearson correlation coefficient primarily measures linear relationships and may not fully capture non-linear relationships or the combined effects of dynamic features.

To more comprehensively analyze the relationship between health factors and SOH, this paper also introduces grey relational analysis. Grey relational analysis is a numerical method based on sequence comparison, which is suitable for evaluating the correlation between feature variables and the target variable (SOH). The grey relational analysis not only evaluates the relative change trends between variables but also more effectively reveals the relative influence and hierarchical relationships between variables, especially under conditions with limited data. The results from grey relational analysis can supplement and validate the Pearson correlation results, providing a more accurate assessment of the impact of each health factor on SOH. The specific steps for grey relational analysis are as follows:

1. Standardize the data: Normalize all data sequences to a common range to facilitate comparison.
2. Calculate the reference sequence: The SOH values are selected as the reference sequence.
3. Calculate the absolute difference sequence: Compute the absolute differences between each feature sequence ($\:{F}_{1\:}$-$\:{F}_{8\:})$and the reference sequence (SOH).
4. Calculate the grey relational coefficient: This coefficient represents the degree of correlation between the reference sequence and each feature sequence, as shown in (24):

24 $$\:\begin{array}{c}{{\upxi\:}}_{ij}=\frac{{{\Delta\:}}_{min}+{\uprho\:}\cdot\:{{\Delta\:}}_{max}}{{{\Delta\:}}_{ij}+{\uprho\:}\cdot\:{{\Delta\:}}_{max}}\end{array}$$

Where $\:{{\Delta\:}}_{min}\:$is the minimum of all absolute differences,$\:\:{{\Delta\:}}_{max}\:$is the maximum of all absolute differences, and$\:\:{{\Delta\:}}_{ij}\:$is the absolute difference of the$\:\:i$-th feature at the$\:\:j$-th time point.$\:\:{\uprho\:}\:$represents the resolution coefficient, generally$\:\:\:{\uprho\:}\in\:\left(\mathrm{0,1}\right)$, in this case$\:\:\:{\uprho\:}=0.5$.

Based on the steps of grey relational analysis above, the correlation between each health factor and the battery’s SOH is obtained, as shown in Table 3. Due to differences in the capacity degradation rates of different battery groups, the extracted health factors reflect their respective variation trends. From Table 3, it can be seen tha$\:{\:\:F}_{3\:}$and$\:\:{\:F}_{7\:}$both show strong correlations with SOH. Essentially, $\:{F}_{3\:}$reflects the duration of the constant current charging phase, while$\:\:{F}_{7\:}$, as the peak of the IC curve, reflects the battery’s dynamic characteristics and internal health state. Therefore, they should exhibit a strong correlation with SOH, and the calculation results validate this. In summary, except for a few features that show weaker correlations in specific batteries, the remaining health factors generally show a high correlation with SOH.

**Table 3:** Grey correlation between health features and SOH.

Battery	Health factors							
	$\:{F}_{1}$	$\:{F}_{2}$	$\:{F}_{3}$	$\:{F}_{4}$	$\:{F}_{5}$	$\:{F}_{6}$	$\:{F}_{7}$	$\:{F}_{8}$
B0005	0.5230	0.5877	0.7713	0.7747	0.5310	0.5026	0.8171	0.5815
B0006	0.9307	0.8540	0.9313	0.5115	0.6991	0.5297	0.8814	0.5528
B0007	0.5304	0.6693	0.9618	0.5518	0.6542	0.5478	0.7781	0.6175
B0018	0.6538	0.6547	0.8540	0.5535	0.6740	0.5688	0.8811	0.6340

To optimize feature utilization for SOH estimation, this study employs a dual-analytical approach combining Pearson correlation coefficient analysis and grey relational analysis to evaluate the relationship between extracted health factors and battery SOH degradation.

As shown in Fig. 2, the Pearson correlation heatmap reveals that most health factors ($\:{F}_{1}$-$\:{F}_{8})$ exhibit strong correlations with SOH, with coefficients generally exceeding 0.5. However, to capture non-linear relationships that may be missed by linear correlation analysis, grey relational analysis is applied as a complementary tool following the standard four-step procedure outlined in Eq. (24).

The comprehensive results in Table 3 demonstrate that $\:{F}_{3}$(constant current charging duration) and $\:{F}_{7}$(IC curve peak value) consistently show the highest correlation values across all battery datasets, with grey correlation coefficients typically exceeding 0.77. These findings validate the effectiveness of the selected health factors in characterizing battery degradation patterns, with $\:{F}_{3}$ reflecting charge acceptance capability and $\:{F}_{7}$ representing dynamic electrochemical characteristics.

While this dual-correlation analysis confirms the relevance of extracted features for SOH estimation by evaluating feature-to-SOH relationships, the correlation structure among features themselves exhibits significant temporal variability throughout battery lifecycle. Section “Temporal evolution analysis of indicator correlations” presents a detailed analysis of how inter-feature correlations evolve across degradation stages using Pearson correlation matrices, providing empirical evidence for the necessity of the adaptive weighting mechanism described in Sect. “Multi-indicator feature weighting block”.

#### Dataset construction

Based on the correlation analysis results, the feature dataset is constructed for subsequent model training. The validated health factors are integrated as model inputs, with their correlation strengths serving as the foundation for the weighting strategy detailed in Sect. “Multi-indicator feature weighting block”. Following standard machine learning practices and considering dataset size constraints, the data is partitioned as follows: the first 60% of cycles are allocated to training and validation sets, while the remaining 40% constitutes the test set. For NASA batteries, this corresponds to approximately 100 cycles for training, while for CALCE batteries, this translates to 530–598 cycles for training. This 60/40 split ensures sufficient training samples while maintaining representative test data to adequately evaluate the model’s generalization capability across different battery degradation scenarios.

### Main experiments

#### Experimental setup

All experiments in this study were conducted on a computer equipped with Windows 11, an NVIDIA RTX 4060 GPU, an Intel i7-12650 CPU, and 32GB of RAM. The programming language used was Python (version 3.9), and the models were built using the TensorFlow backend (version 2.6.0) and the Keras library (version 1.1.2). The main parameters of the MI-SOH model are shown in Table 4. (Note: ‘lb’ denotes the lower bound of the parameter range, and ‘ub’ denotes the upper bound of the parameter range)

**Table 4:** The parameter settings of the MI-SOH model.

Parameters	Values
TCN hidden channels	[lb: 16, ub: 128]
TCN kernel size	[lb: 2, ub: 5]
TCN dilation	[1, 2, 4, 8]
iTransformer d_model	[lb: 32, ub: 256]
iTransformer nhead	[lb: 1, ub: 8]
iTransformer num_layers	[lb: 1, ub: 4]
Learning rate	[lb: 0.0001, ub: 0.01]
Batch size	[8, 16, 32, 64]
Training epoch	50
Optimizer	Adam

For the multi-indicator feature weighting mechanism (Sect. “Multi-indicator feature weighting block”), Pearson correlation coefficients and grey relational degrees are computed on the training dataset, which encompasses cycles across multiple degradation stages (approximately 60% of total lifecycle, covering SOH from 100% to ~ 75%). This multi-stage training data ensures that the learned weights $\:{w}_{i}$ (Eq. 2) reflect the time-varying importance patterns of health indicators throughout battery aging. While feature weights remain fixed during inference for computational efficiency, they inherently adapt to temporal variability by being trained on data representing diverse degradation contexts, as validated by the correlation evolution analysis in Sect. “Temporal evolution analysis of indicator correlations”.

#### Evaluation metrics

To accurately evaluate the performance of the proposed estimation model, we use Root Mean Squared Error (RMSE), Mean Squared Error (MSE), Mean Absolute Error (MAE), Mean Absolute Percentage Error (MAPE), and the Coefficient of Determination (R²) as performance evaluation metrics. These measures reflect the accuracy and effectiveness of the model’s estimation from different perspectives. The formulas for calculating these evaluation metrics are shown in (25) to (29).

25 $$\:\begin{array}{c}RMSE=\sqrt{\frac{1}{n}{\sum\:}_{i=1}^{n}{\left({y}_{p}-{y}_{i}\right)}^{2}}\end{array}$$

26 $$\:\begin{array}{c}MSE=\frac{1}{n}{\sum\:}_{i=1}^{n}{\left({y}_{p}-{y}_{i}\right)}^{2}\end{array}$$

27 $$\:\begin{array}{c}MAE=\frac{1}{n}{\sum\:}_{i=1}^{n}\left|{y}_{p}-{y}_{i}\right|\end{array}$$

28 $$\:\begin{array}{c}MAPE=\frac{1}{n}{\sum\:}_{i=1}^{n}\left|\frac{\overline{{y}_{i}}-{y}_{i}}{{y}_{i}}\right|\times\:100\%\end{array}$$

29 $$\:\begin{array}{c}{R}^{2}=1-\frac{{\sum\:}_{i=1}^{n}{\left({y}_{p}-{y}_{i}\right)}^{2}}{{\sum\:}_{i=1}^{n}{\left(\overline{{y}_{i}}-{y}_{i}\right)}^{2}}\end{array}$$

Where $\:n$ represents the number of samples, $\:{y}_{p}\:$is the model’s predicted result, $\:{y}_{i}\:$is the actual SOH value, and $\:\overline{{y}_{i}}\:$is the mean value.

#### Hyperparameter optimization comparison

To validate the ZOA selection rationale introduced in Sect. “Introduction”, we conduct a comprehensive empirical comparison of four metaheuristic algorithms on the B0005 battery dataset: PSO^19^, WOA^20^, GWO^21^, and ZOA^22^.

Experimental Configuration: All algorithms employ identical settings to ensure fair comparison: population size of 10, maximum iterations of 60, and 5-fold time-series cross-validation (TimeSeriesSplit) for fitness evaluation on the validation set. The hyperparameter search space follows Table 4. We employ TimeSeriesSplit rather than standard K-fold cross-validation to prevent temporal data leakage, as random shuffling violates the causal structure of battery degradation sequences^51^.

**Fig. 10:**
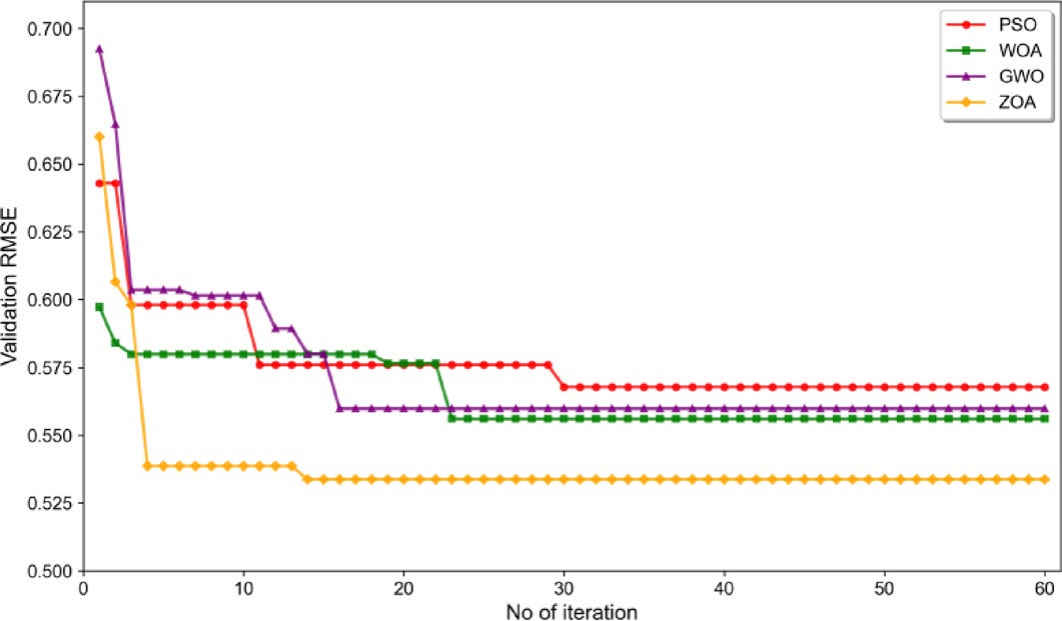
Convergence curves of four metaheuristic algorithms.

Results and Analysis: As shown in Table 5, for TCN-iTransformer architecture optimization, ZOA achieves the lowest validation RMSE of 0.5338 on B0005, outperforming GWO (0.5598, + 4.9%), WOA (0.5561, + 4.2%), and PSO (0.5679, + 6.4%). Figure 10 illustrates the convergence trajectories, where ZOA demonstrates rapid early-stage convergence (iteration 4: 0.5386) followed by sustained refinement (iteration 14: 0.5338), while PSO exhibits the slowest convergence (iteration 30: 0.5679) and WOA stagnates prematurely at 0.5561 despite fast initial descent. GWO shows competitive performance (0.5598), converging at iteration 16 through its hierarchical alpha-beta-delta structure.

**Table 5:** TCN-iTransformer hyperparameter optimization algorithm comparison.

Algorithm	Best RMSE	Initial RMSE	Convergence Iter.	Time (min)
PSO	0.5679	0.6429	30	43.63
WOA	0.5561	0.5973	23	32.59
GWO	0.5598	0.6926	16	34.25
**ZOA**	**0.5338**	**0.6601**	**14**	**30.09**

The results validate ZOA’s theoretical advantage: its dual-phase foraging-defense mechanism achieves superior exploration-exploitation balance compared to PSO’s fixed inertia weight and WOA’s probabilistic switching. ZOA’s 19.1% improvement from initial population (0.6601→0.5338) exceeds PSO (11.7%) and WOA (6.9%), while approaching GWO (19.2%) with faster convergence (14 vs. 16 iterations). Regarding computational efficiency, ZOA completes optimization in 30.1 min, faster than WOA (32.6 min), GWO (34.3 min), and significantly faster than PSO (43.6 min), demonstrating optimal balance between solution quality and computational cost.

The identified optimal configuration serves as the foundation for all subsequent model comparison experiments.

#### Comparison experiment

To comprehensively evaluate MI-SOH’s performance and generalization capability, comparative experiments are conducted on both NASA and CALCE datasets. Eight health indicators are used as model inputs, with battery capacity as the output target. The MI-SOH model is compared against three baseline approaches: TCN, iTransformer, and TCN-iTransformer.

**Fig. 11:**
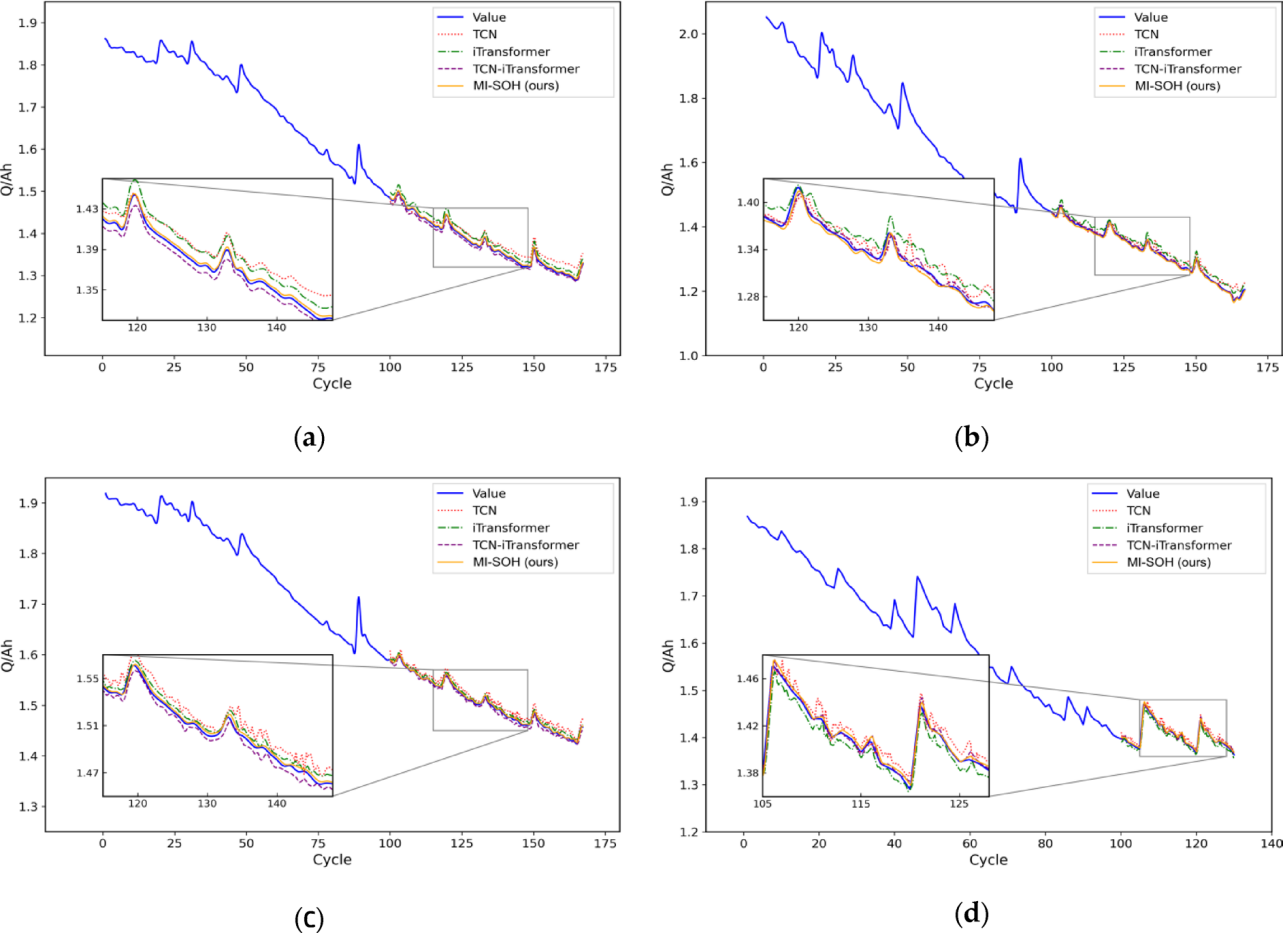
The comparison experiment results of four lithium-ion batteries on the NASA dataset. **(a)** B0005. **(b)** B0006. **(c)** B0007. **(d)** B0018.

**Fig. 12:**
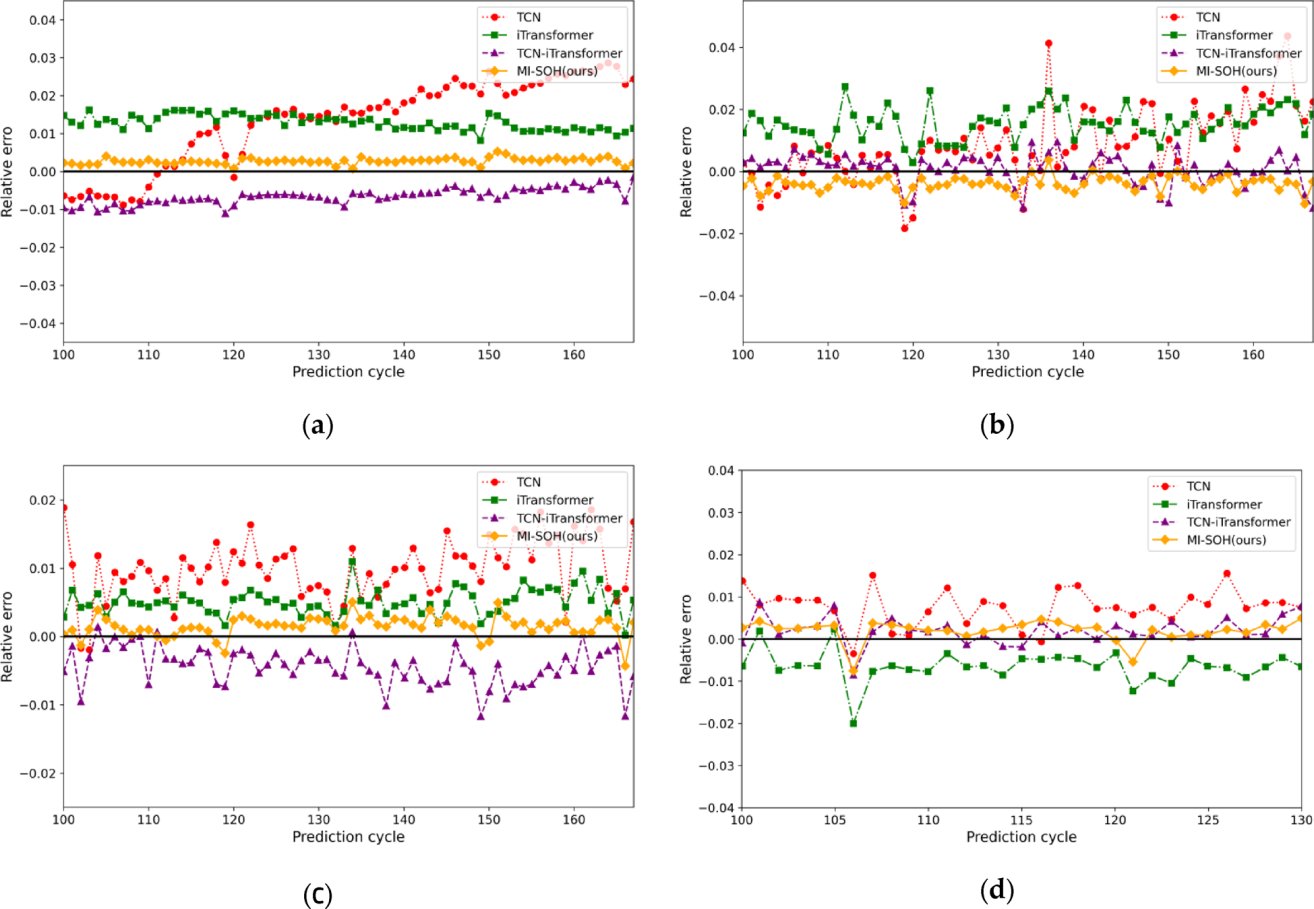
The experimental error results of four lithium-ion batteries on the NASA dataset. **(a)** B0005. **(b)** B0006. **(c)** B0007. **(d)** B0018.

Figure 11 presents SOH estimation results for NASA batteries (B0005, B0006, B0007, B0018), with corresponding relative errors shown in Fig. 12. The estimation curves reveal substantial performance differences across models. Single-architecture models (TCN, iTransformer) exhibit significant deviations from actual degradation trajectories, particularly during capacity recovery phenomena where accumulated errors compromise stability. The TCN-iTransformer fusion model demonstrates improved performance by integrating temporal convolutions with cross-variable attention, achieving closer alignment with ground truth. However, it remains suboptimal due to fixed hyperparameters limiting its adaptability. The proposed MI-SOH model achieves superior accuracy through adaptive multi-indicator weighting and ZOA-optimized hyperparameters, effectively capturing both local temporal patterns and long-term dependencies across diverse aging scenarios.

**Fig. 13:**
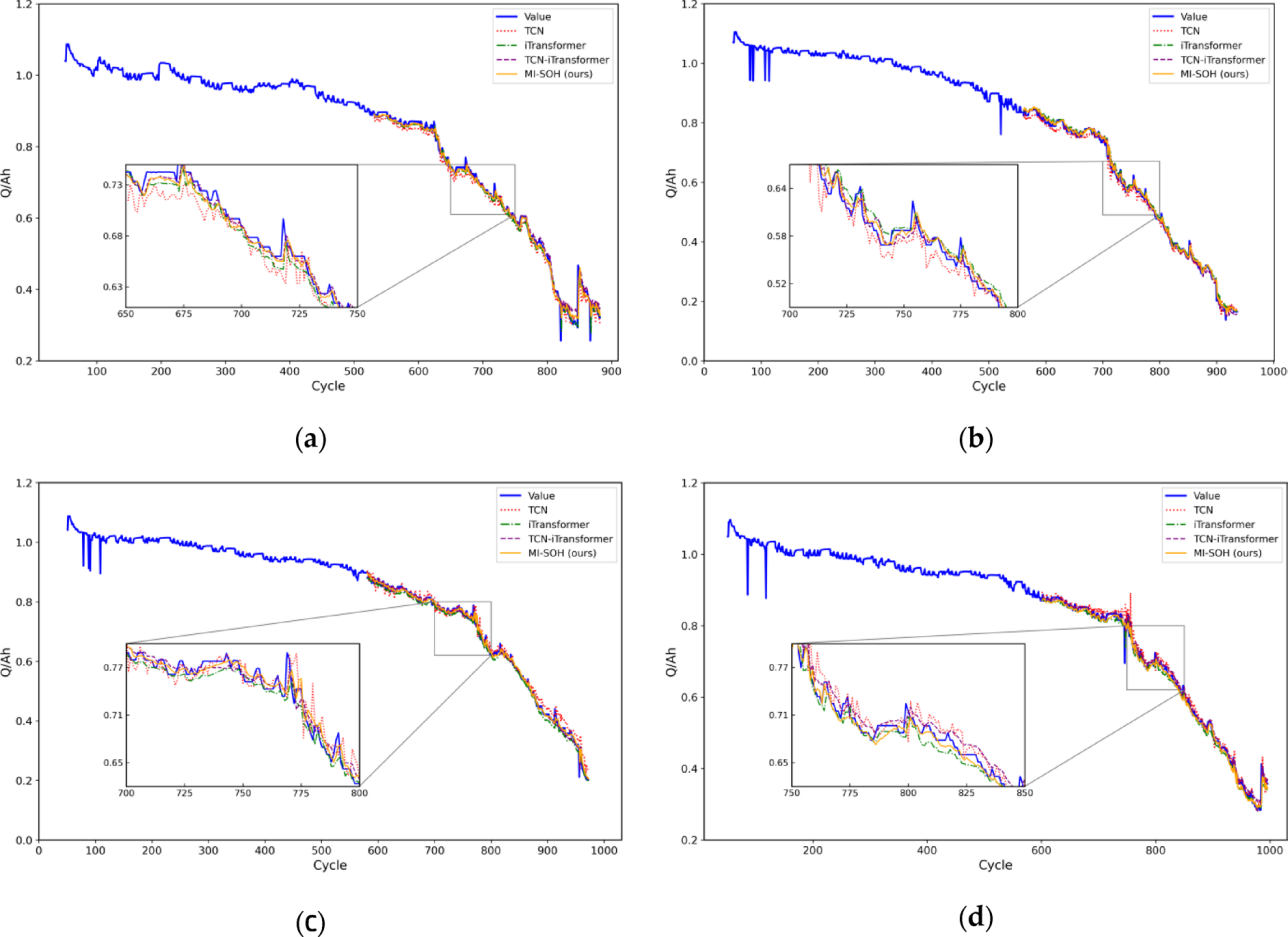
The comparison experiment results of four lithium-ion batteries on the CALCE dataset. **(a)** CS2_35. **(b)** CS2_36. **(c)** CS2_37. **(d)** CS2_38.

**Fig. 14:**
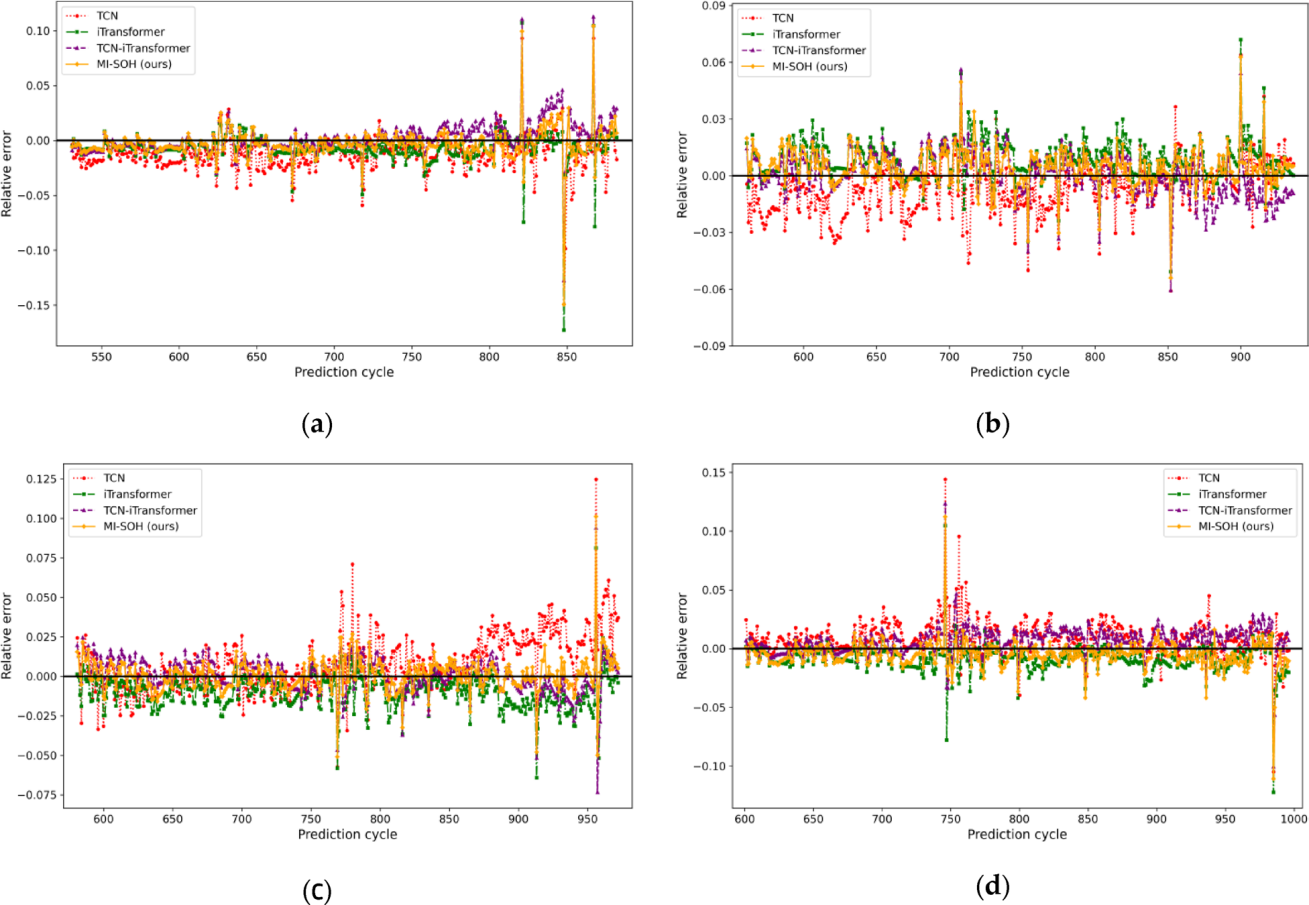
The experimental error results of four lithium-ion batteries on the CALCE dataset. **(a)** CS2_35. **(b)** CS2_36. **(c)** CS2_37. **(d)** CS2_38.

To validate cross-chemistry generalization, experiments are extended to CALCE batteries (CS2_35, CS2_36, CS2_37, CS2_38) featuring substantially longer lifecycles (882–996 cycles) and distinct LCO chemistry. Figures 13 and 14 illustrate estimation results and relative errors, respectively. Despite the extended degradation timescales and different electrochemical characteristics, MI-SOH maintains consistent superiority over baseline methods. The model successfully adapts to CALCE’s gradual degradation patterns, demonstrating robustness across battery chemistries and form factors. Notably, all models exhibit slightly higher RMSE values on CALCE compared to NASA (Table 6), attributed to increased data complexity from prolonged cycling and multi-stage degradation transitions inherent in LCO prismatic cells.

Table 6 presents comprehensive performance metrics across all eight batteries, revealing MI-SOH’s consistent advantages. On NASA dataset, MI-SOH achieves average improvements of 75.24% (RMSE) and 76.67% (MAE) over TCN, 70.26% (RMSE) and 74.19% (MAE) over iTransformer, and 39.18% (RMSE) and 42.18% (MAE) over TCN-iTransformer. On CALCE dataset, the improvements are 34.53% (RMSE) and 42.43% (MAE) over TCN, 18.93% (RMSE) and 25.05% (MAE) over iTransformer, and 9.12% (RMSE) and 14.32% (MAE) over TCN-iTransformer. The cross-dataset performance summary in Table 7 demonstrates MI-SOH’s consistent superiority, achieving average RMSE of 0.00312 (NASA) and 0.01126 (CALCE) with average R² of 0.996 on both datasets.

The consistent performance improvements across NASA (NCA cylindrical, short lifecycle) and CALCE (LCO prismatic, extended lifecycle) datasets validate MI-SOH’s robust generalization capability. The adaptive weighting mechanism successfully accommodates diverse correlation evolution patterns across different chemistries, while ZOA optimization ensures optimal hyperparameter configurations for each battery type. These results confirm that the proposed framework effectively addresses the fundamental challenge of transferring SOH estimation models across heterogeneous battery technologies.

**Table 6:** Performance comparison across eight lithium-ion batteries from NASA and CALCE datasets.

Battery	Models	RMSE	MSE	MAE	MAPE	*R* ^2^
B0005	TCN	0.01749	0.000306	0.01558	0.01156	0.907
	iTransformer	0.01296	0.000168	0.01280	0.00928	0.949
	TCN-iTransformer	0.00682	0.000047	0.00651	0.00469	0.986
	**MI-SOH (ours)**	**0.00283**	**0.000008**	**0.00271**	**0.00198**	**0.998**
B0006	TCN	0.01426	0.000203	0.01128	0.00882	0.967
	iTransformer	0.01660	0.000276	0.01559	0.01190	0.955
	TCN-iTransformer	0.00498	0.000025	0.00407	0.00308	0.995
	**MI-SOH (ours)**	**0.00463**	**0.000021**	**0.00383**	**0.00290**	**0.996**
B0007	TCN	0.01091	0.000119	0.00999	0.00668	0.949
	iTransformer	0.00529	0.000028	0.00492	0.00328	0.988
	TCN-iTransformer	0.00515	0.000027	0.00439	0.00294	0.989
	**MI-SOH (ours)**	**0.00212**	**0.000005**	**0.00183**	**0.00122**	**0.998**
B0018	TCN	0.00774	0.000060	0.00686	0.00490	0.904
	iTransformer	0.00710	0.000050	0.00619	0.00440	0.920
	TCN-iTransformer	0.00357	0.000013	0.00267	0.00190	0.980
	**MI-SOH (ours)**	**0.00289**	**0.000008**	**0.00183**	**0.00130**	**0.992**
CS2_35	TCN	0.01965	0.000386	0.01475	0.02865	0.987
	iTransformer	0.01565	0.000245	0.00922	0.01868	0.992
	TCN-iTransformer	0.01462	0.000214	0.00879	0.02051	0.993
	**MI-SOH (ours)**	**0.01377**	**0.000190**	**0.00809**	**0.01762**	**0.994**
CS2_36	TCN	0.01442	0.000208	0.01127	0.02584	0.995
	iTransformer	0.01237	0.000153	0.00948	0.02346	0.996
	TCN-iTransformer	0.01091	0.000119	0.00828	0.02233	0.997
	**MI-SOH (ours)**	**0.00976**	**0.000095**	**0.00698**	**0.01681**	**0.998**
CS2_37	TCN	0.01819	0.000331	0.01338	0.03229	0.989
	iTransformer	0.01375	0.000189	0.01069	0.02213	0.994
	TCN-iTransformer	0.01097	0.000120	0.00781	0.01644	0.996
	**MI-SOH (ours)**	**0.00982**	**0.000096**	**0.00657**	**0.01375**	**0.997**
CS2_38	TCN	0.01655	0.000274	0.01131	0.02068	0.991
	iTransformer	0.01380	0.000190	0.00958	0.01821	0.993
	TCN-iTransformer	0.01305	0.000170	0.00920	0.01875	0.994
	**MI-SOH (ours)**	**0.01169**	**0.000137**	**0.00756**	**0.01476**	**0.995**

**Table 7:** Average performance comparison across NASA and CALCE datasets.

Dataset	Models	Avg. RMSE	Avg. MAE	Avg. *R*^2^
NASA	TCN	0.01260	0.01093	0.932
	iTransformer	0.01049	0.00988	0.953
	TCN-iTransformer	0.00513	0.00441	0.988
	**MI-SOH (ours)**	**0.00312**	**0.00255**	**0.996**
CALCE	TCN	0.01720	0.01268	0.991
	iTransformer	0.01389	0.00974	0.994
	TCN-iTransformer	0.01239	0.00852	0.995
	**MI-SOH (ours)**	**0.01126**	**0.00730**	**0.996**

### Temporal evolution analysis of indicator correlations

To validate the fundamental premise of MI-SOH’s adaptive weighting mechanism, we systematically analyze how correlations between health indicators evolve throughout battery degradation. This analysis provides empirical evidence demonstrating why static feature fusion strategies are fundamentally inadequate for accurate SOH estimation.

**Fig. 15:**
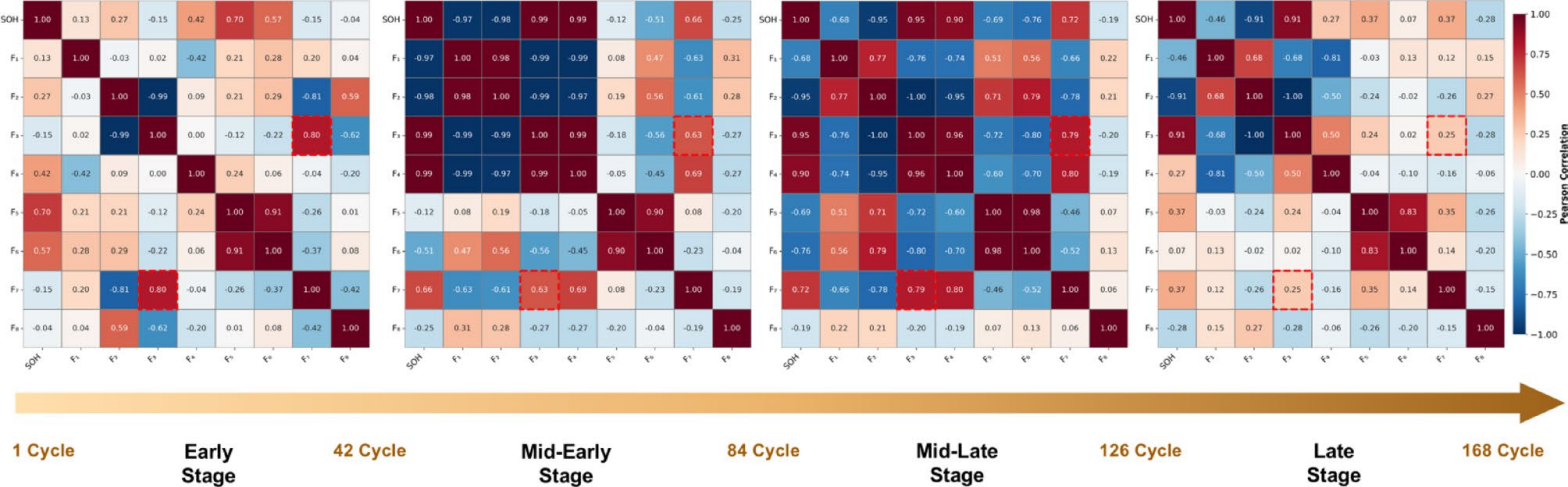
Temporal evolution of indicator correlations across four degradation stages for B0005 battery. Red dashed boxes highlight key indicator pairs demonstrating dramatic correlation changes.

We partition the B0005 battery’s 168 charge-discharge cycles into four degradation stages based on SOH thresholds: Early (Cycles 1–42, SOH > 95%), Mid-Early (43–84, 85%< SOH ≤ 95%), Mid-Late (85–126, 75%< SOH ≤ 85%), and Late (127–168, SOH ≤ 75%). For each stage, we compute Pearson correlation matrices among the eight health indicators, revealing striking temporal variability in indicator interdependencies, as illustrated in Fig. 15.

**Diverse Correlation Evolution Patterns**: The correlation structure exhibits substantial temporal variability, with three distinct evolutionary patterns emerging from our analysis. First, *correlation weakening* reflects mechanism decoupling: the correlation between $\:{F}_{3\:}$(constant current charging duration) and $\:{F}_{7\:}$(IC peak value) decreases dramatically from 0.803 in early stages to 0.255 in late stages—a 68% reduction. This phenomenon occurs because $\:{F}_{3}$ becomes increasingly dominated by resistance growth while $\:{F}_{7\:}$primarily reflects active material loss, causing these mechanisms to evolve independently in late stages. Second, *correlation strengthening and reversal* indicates emerging couplings: voltage-related indicator pairs ($\:{F}_{1\:}$-$\:{F}_{2}$) shift from weak negative correlation (− 0.033) to strong positive correlation (0.677) as internal resistance increases and polarization effects intensify. Third, *non-monotonic evolution* demonstrates complex temporal dynamics: the $\:{F}_{3\:}$-$\:{F}_{7\:}$trajectory (0.803→0.627→0.790→0.255) reflects competing degradation mechanisms with distinct activation patterns throughout battery lifecycle.

**Implications for Adaptive Weighting**: These findings reveal a fundamental challenge for existing methods: the optimal weighting of health indicators is not static but varies dramatically across battery lifecycle. Methods employing fixed feature importance cannot accommodate (1) bidirectional changes where some correlations strengthen while others weaken, (2) polarity reversals where pairs transition from negative to positive correlation, and (3) non-monotonic evolution requiring context-aware adaptation. The MI-SOH framework addresses these challenges through dual-correlation adaptive weighting (Sect. “Multi-indicator feature weighting block”), which dynamically reweights features based on both linear and non-linear dependencies. This mechanism enables automatic adjustment to evolving indicator importance throughout battery aging. The necessity of adaptive weighting to accommodate these temporal dynamics is further validated in Sect. “Ablation study”.

### Ablation study

To validate the effectiveness of each fundamental component in the proposed MI-SOH model, we conducted comprehensive ablation experiments by systematically removing or replacing key modules. four variants were designed to assess the contribution of individual components:

#### w/o weighting:

The direct weighting method based on Pearson correlation and grey relational analysis is removed, using equal weights for all health factors instead.

#### w/o TCN:

The TCN component is eliminated, feeding preprocessed features directly to the iTransformer module to evaluate the contribution of temporal convolutional operations.

#### w/o iTransformer:

The iTransformer module is removed, using only TCN followed by a linear layer for SOH estimation to assess the importance of cross-variable attention mechanisms.

#### w/o ZOA:

The ZOA optimization algorithm is replaced with manual hyperparameter tuning, using grid search to determine optimal parameters for the TCN-iTransformer model.

The complete proposed MI-SOH model incorporating all components including multi-feature weighting, TCN temporal modeling, iTransformer cross-variable attention, and ZOA hyperparameter optimization.

The experimental results presented in Table 8 demonstrate the significant contribution of each component to the overall model performance. The complete MI-SOH model achieves the best average performance across all evaluation metrics with average RMSE of 0.00312, average MSE of 0.000011, average MAE of 0.00255, average MAPE of 0.00185, and average R² of 0.996.

**Table 8:** Ablation study results showing the contribution of each component in the proposed model (averaged across B0005, B0006, B0007, and B0018 datasets).

Models	RMSE	MSE	MAE	MAPE	*R* ^2^
w/o Weighting	0.00589	0.000035	0.00518	0.00379	0.985
w/o TCN	0.00725	0.000053	0.00642	0.00458	0.978
w/o iTransformer	0.00698	0.000049	0.00615	0.00435	0.981
w/o ZOA	0.00513	0.000028	0.00441	0.00315	0.988
**MI-SOH (ours)**	**0.00312**	**0.000011**	**0.00255**	**0.00185**	**0.996**

Removing the weighting mechanism (w/o Weighting) results in performance degradation with average RMSE increasing to 0.00589, demonstrating that the combined Pearson correlation and grey relational analysis effectively identifies and emphasizes the most relevant health factors. This substantial performance drop directly validates the findings in Sect. “Temporal evolution analysis of indicator correlations” when the model treats all indicators with equal importance despite their time-varying correlations—such as the$\:\:{F}_{3}$-$\:{F}_{7\:}$pair that weakens from 0.803 to 0.255 across degradation stages—estimation accuracy suffers significantly. The adaptive weighting mechanism addresses this challenge by incorporating multi-stage correlation patterns, enabling the model to automatically adjust emphasis across different aging contexts.

The elimination of TCN (w/o TCN) shows substantial impact with average RMSE rising to 0.00725, confirming that temporal convolutional operations are crucial for capturing local temporal dependencies in battery degradation patterns.The removal of iTransformer (w/o iTransformer) leads to average RMSE of 0.00698, indicating that cross-variable attention mechanisms significantly contribute to modeling multivariate correlations among health factors. Most notably, without ZOA optimization (w/o ZOA), the model performance decreases considerably with average RMSE reaching 0.00513, validating the effectiveness of the zebra optimization algorithm in finding optimal hyperparameter configurations.

These ablation results collectively confirm that each proposed component contributes meaningfully to the overall model performance, with the complete integration achieving superior SOH estimation accuracy compared to any individual component removal.

### Complexity analysis

The computational efficiency of SOH estimation models is crucial for real-world battery management applications. We analyze the theoretical and practical computational complexity of the proposed MI-SOH model compared to baseline methods across multiple dimensions.

#### Theoretical complexity analysis:

Table 9 presents the training and inference time complexity for different model architectures. The proposed MI-SOH model has a training time complexity of O(P·I·(L·C·k·log₂L + N²·d)·E), where P represents population size in ZOA, I denotes iterations, L is sequence length, C is channel number, k is kernel size, N is feature dimension, d is model dimension, and E represents training epochs. The inference time complexity is O(L·C·k·log₂L + N²·d), which is competitive with existing methods while providing superior accuracy.

**Table 9:** Computational complexity comparison of different model architectures for lithium-ion battery SOH estimation.

Models	Training Time Complexity	Inference Time Complexity
TCN	O(L·C·k·log₂L·E)	O(L·C·k·log₂L)
Transformer	O(L²·d·E)	O(L²·d)
iTransformer	O(N²·d·E)	O(N²·d)
TCN-Transformer	O((L·C·k·log₂L + L²·d)·E)	O(L·C·k·log₂L + L²·d)
TCN-iTransformer	O((L·C·k·log₂L + N²·d)·E)	O(L·C·k·log₂L + N²·d)
**MI-SOH (ours)**	**O(P·I·(L·C·k·log₂L + N²·d)·E)**	**O(L·C·k·log₂L + N²·d)**

Compared to traditional approaches, the TCN component contributes O(L·C·k·log₂L) complexity for temporal modeling, while the iTransformer adds O(N²·d) for cross-variable attention. The ZOA optimization introduces additional overhead during training but does not affect inference complexity, making the model suitable for real-time applications.

#### Parameter efficiency:

The MI-SOH model maintains reasonable parameter counts while achieving state-of-the-art performance. The multi-feature weighting mechanism adds minimal parameters, while the TCN-iTransformer fusion architecture efficiently balances model capacity with computational requirements. The ZOA optimization helps identify optimal configurations that maximize performance without unnecessary parameter inflation.

#### Training and inference performance:

Experimental results demonstrate that despite the sophisticated architecture, the MI-SOH model maintains practical training and inference times. The parallel processing capabilities of TCN layers combined with the efficient attention mechanisms in iTransformer enable scalable deployment across different battery management scenarios.

The complexity analysis confirms that the proposed MI-SOH model achieves an optimal balance between computational efficiency and predictive accuracy, making it suitable for both offline training and real-time SOH estimation in practical battery management systems.

## Conclusions

To improve the SOH estimation accuracy of lithium-ion batteries, This paper presents MI-SOH (Multi-indicator Enhanced Temporal Adaptive Learning), a comprehensive framework that systematically integrates multi-indicator feature weighting, temporal pattern extraction, and adaptive hyperparameter optimization to address critical limitations in existing SOH estimation methods.

First, the moving average method and cubic spline interpolation method were applied to solve the noise and fluctuation problems in traditional data preprocessing. These techniques effectively improved the data quality and provided a reliable foundation for subsequent modeling. Secondly, based on the feature evaluation method combining Pearson correlation coefficient and grey correlation degree analysis, key health factors such as the constant current charging duration and IC curve peak value, which show a high correlation with SOH (correlation degree > 0.77), were identified and validated. Furthermore, temporal evolution analysis (Sect. “Temporal evolution analysis of indicator correlations”) revealed that indicator correlations undergo diverse transformations across degradation stages—with certain pairs weakening by 68% while others strengthen significantly—validating the necessity of the adaptive weighting mechanism that reflects multi-stage degradation patterns rather than employing static feature importance throughout battery lifecycle. A direct weighting method was used to highlight the contribution of these highly correlated features in the model training process. Thirdly, the ZOA optimization algorithm was introduced to globally optimize the hyperparameters of the TCN–iTransformer model, effectively solving the inefficiency problem of traditional optimization methods in the parameter tuning process. Finally, experimental validation on NASA and CALCE datasets demonstrates that the MI-SOH model achieves average RMSE of 0.00312 and 0.01126 respectively, with average R² of 0.996 across both datasets, fully verifying the effectiveness and reliability of the proposed method in improving SOH estimation accuracy across diverse lithium-ion battery chemistries and lifecycles. Ablation studies confirm each component’s meaningful contribution, while computational complexity analysis validates practical feasibility with O(L·C·k·log₂L + N²·d) inference complexity suitable for real-time applications.

Despite the significant performance demonstrated, certain limitations warrant future investigation. Future research should focus on three key directions. First, integrating MI-SOH into electric vehicle battery management systems for industrial deployment. Second, inspired by recent advances in scalable battery health prognosis^23,24^, exploring computational scalability enhancements through structured sparse approximation techniques to efficiently process large-scale datasets as EV fleets expand. Third, incorporating uncertainty quantification mechanisms to provide probabilistic confidence intervals for SOH predictions^25,26^, supporting risk-aware maintenance scheduling and informed battery replacement strategies. These enhancements will strengthen the framework’s applicability in resource-constrained real-world deployments.

The MI-SOH framework establishes a robust foundation for next-generation battery health monitoring in energy systems, demonstrating that systematic multi-indicator analysis and intelligent optimization can significantly advance lithium-ion battery SOH estimation accuracy and reliability. This advancement directly supports the reliable operation of electric vehicles and grid-scale energy storage systems, contributing to the broader goal of sustainable energy transition and carbon neutrality. The improved prediction accuracy reduces unexpected battery failures, extends battery lifespan, and enhances the economic feasibility of electric mobility and renewable energy integration.

## Data Availability

The datasets used in this study are publicly available: NASA battery dataset: https:/ti.arc.nasa.gov; CALCE battery dataset: https:/calce.umd.edu/battery-data.
